# Robust and ultrafast fiducial marker correspondence in electron tomography by a two-stage algorithm considering local constraints

**DOI:** 10.1093/bioinformatics/btaa1098

**Published:** 2021-01-08

**Authors:** Renmin Han, Guojun Li, Xin Gao

**Affiliations:** Research Center for Mathematics and Interdisciplinary Sciences, Shandong University, Qingdao 266237, China; King Abdullah University of Science and Technology (KAUST), Computational Bioscience Research Center (CBRC), Computer, Electrical and Mathematical Sciences and Engineering (CEMSE) Division, Thuwal 23955-6900, Saudi Arabia; Research Center for Mathematics and Interdisciplinary Sciences, Shandong University, Qingdao 266237, China; King Abdullah University of Science and Technology (KAUST), Computational Bioscience Research Center (CBRC), Computer, Electrical and Mathematical Sciences and Engineering (CEMSE) Division, Thuwal 23955-6900, Saudi Arabia

## Abstract

**Motivation:**

Electron tomography (ET) has become an indispensable tool for structural biology studies. In ET, the tilt series alignment and the projection parameter calibration are the key steps toward high-resolution ultrastructure analysis. Usually, fiducial markers are embedded in the sample to aid the alignment. Despite the advances in developing algorithms to find correspondence of fiducial markers from different tilted micrographs, the error rate of the existing methods is still high such that manual correction has to be conducted. In addition, existing algorithms do not work well when the number of fiducial markers is high.

**Results:**

In this article, we try to completely solve the fiducial marker correspondence problem. We propose to divide the workflow of fiducial marker correspondence into two stages: (i) initial transformation determination, and (ii) local correspondence refinement. In the first stage, we model the transform estimation as a correspondence pair inquiry and verification problem. The local geometric constraints and invariant features are used to reduce the complexity of the problem. In the second stage, we encode the geometric distribution of the fiducial markers by a weighted Gaussian mixture model and introduce drift parameters to correct the effects of beam-induced motion and sample deformation. Comprehensive experiments on real-world datasets demonstrate the robustness, efficiency and effectiveness of the proposed algorithm. Especially, the proposed two-stage algorithm is able to produce an accurate tracking within an average of  ⩽ 100 ms per image, even for micrographs with hundreds of fiducial markers, which makes the real-time ET data processing possible.

**Availability and implementation:**

The code is available at https://github.com/icthrm/auto-tilt-pair. Additionally, the detailed original figures demonstrated in the experiments can be accessed at https://rb.gy/6adtk4.

**Supplementary information:**

Supplementary data are available at *Bioinformatics* online.

## 1 Introduction

Electron tomography (ET) is a powerful and indispensable tool to solve the three-dimensional (3D) ultrastructure (Frank, 2006), by reconstructing from a series of micrographs (tilt series) taken with different tilt angles. The development of ET has bridged the resolution gap between cellular imaging and high-resolution ultrastructure analysis (Rigort et al., 2010; Wan and Briggs, 2016). Especially, the recent application of ET in subtomogram averaging has advanced the limits of *in situ* ultrastructure analysis, which may lead to the next revolution in structural biology (Himes and Zhang, 2018).

The reconstruction of a high-quality ultrastructure relies on the consistency between the 3D projection model and the real-world projections. Before reconstruction, an accurate tilt series alignment is required. Usually, gold beads are used as fiducial markers to assist the alignment (Brandt and Ziese, 2006; Castaño-Díez et al., 2007; Kremer et al., 1996; Lawrence, 1992). So far, fiducial marker-based alignment is still the most widely used alignment method in high-resolution ET (Mastronarde and Held, 2017).

Finding the correspondence of the fiducial markers on different tilted micrographs is the first and most important step in tilt series alignment. After many years of effort, several automated methods have been proposed: RAPTOR (Amat et al., 2008) uses the Markov random field (MRF) to encode the positions of fiducial markers and utilizes the Loopy belief propagation theory to establish the correspondence. The defect of this method is the high computational cost in building the probabilistic model and the high failure rate. Later, Han et al. (2015) utilizes a random sampling method to determine the correspondence. Though the run time has been significantly reduced compared with RAPTOR, the method still faces an increasing computational cost when the number of fiducial markers increases. IMOD makes an automatization for its alignment workflow by first detecting the fiducial markers from the low tilt micrograph and then propagating the correspondence with a nearest neighbor search (Mastronarde and Held, 2017). Nevertheless, a pre-alignment of the tilt series is necessary and the method is not applicable when a significant transformation happens. With the proof of the error bound in a fiducial marker tracking model, a Gaussian mixture model (GMM) based fast-tracking method is proposed (Han et al., 2018). In the fast-tracking model, the correspondence is determined by minimizing the Bayesian probability in fiducial marker assignment. However, the method is not robust to the undesired outliers and relatively large transformation. Therefore, a robust, accurate and automatic fiducial marker correspondence is still one of the scientific challenges in the field (Jensen, 2019).

Recently, non-linear alignment and reconstruction have been further proposed in ET (Fernandez et al., 2018; Lawrence et al., 2006). According to the most recent research, sample deformation and beam-induced motion have been underestimated (Fernandez *et al.*, 2018; Zheng et al., 2017). Fernandez et al. (2019)’s work has clearly demonstrated the warping during sample tilt and how the deformation correction benefits the reconstruction. However, there are only very few works that take the effect of non-uniform distortion into consideration in fiducial marker tracking.

In this article, we propose to divide the determination of fiducial marker correspondence into two stages: (i) initial transformation determination and (ii) local correspondence refinement. A novel two-stage algorithm by considering the local geometric constraint is proposed to ensure the efficient fiducial marker tracking while keeping robustness and accuracy. In the first stage, we model the initial transformation estimation as a correspondence pair inquiry and verification problem. The local invariant features are used for the fast comparison of the geometric similarity between different fiducial marker positions on different micrographs. Within each similarity inquiry, we limit the range of local feature extraction and propose a multiple-check technique of the extracted features for fast consistency verification. In the second stage, we encode the geometric distribution of the fiducial markers by a weighted Gaussian mixture model and solve the correspondence of distributions by an expectation-maximization algorithm, where the parameters about non-uniform drift are introduced to correct the potential distortion. The obvious outliers are pre-excluded based on the solved initial transformation.

With the two-stage algorithm, our aim is to completely solve the fiducial marker correspondence and tracking problem in ET. The utilization of local geometric constraints further reduces the computational complexity and the introduction of drift parameters ensures the accuracy in fiducial marker correspondence determination. Comprehensive experiments on real-world datasets demonstrate the robustness, efficiency and effectiveness of the proposed algorithm. Especially, the proposed algorithm is able to produce an accurate tracking within an average of  ⩽ 100 ms per image, even for micrographs with hundreds of fiducial markers, which is more than 40× faster than the state-of-the-art methods, while achieving ∼99% detection accuracy.

## 2 Materials and methods

### 2.1 Problem formulation

First, we would like to redefine the problem under the terminology of point set registration:

Denoting the positions of fiducial markers from a micrograph as the fixed ‘scene’ point set $X={\left\{x_{n}\right\}}_{n=1,\ldots ,N}$ and the positions of fiducial markers from another micrograph as the moving ‘model’ point set $Y={\left\{y_{m}\right\}}_{m=1,\ldots ,M}$, the problem of the correspondence determination is to find a transform T(·) so that there is a subset of T(Y) with the maximum cardinality that aligns the points from a subset of the fixed ‘scene’ set X under a selected measure of distance or similarity.

**Fig. 1.**
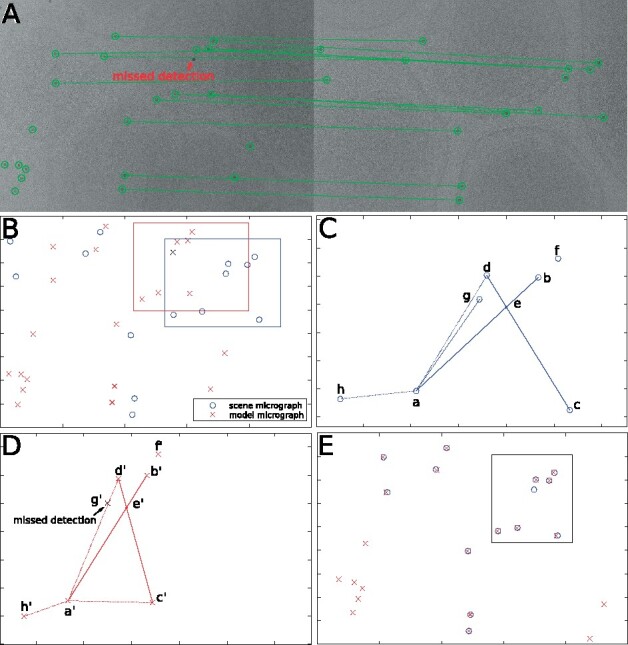
(**A**) The detected fiducial markers and correspondences between two micrographs, where the left is with a high tilt angle and the right is with a low tilt angle. (**B**) Superimposition of fiducial marker positions extracted from the micrographs. (**C**) The geometric constraint within the blue rectangle. (**D**) The geometric constraint within the red rectangle. (**E**) Superimposition of fiducial marker positions after an affine transformation has been applied to the ‘model’ points

Figure 1 gives a schematic illustration of the determination of fiducial marker correspondence between two tilted micrographs.

### 2.2 Fast estimation of initial transformation

The fiducial marker correspondence upon micrographs with different tilt angles approximately follows an affine relationship (Han *et al.*, 2018). For a single point $p={\left[x,y\right]}^{T}$, the affine transformation is defined as

(1) T(p′;A,t)=Ap+t,

where ***A*** is a 2 × 2 affine matrix and ***t*** is a 2 × 1 translation vector. Without considering the sample deformation, an affine transformation is able to describe the correspondence of the fiducial markers extracted from two different tilted micrographs (Fig. 1E). Given a ‘scene’ point set with *N* points and a ‘model’ point set with *M* points, the time complexity is $O(M^{3}N^{3})$ for a baseline method (Three pairs of corresponding points are needed for the estimation of an affine transformation with six free parameters. A simple algorithm to find three corresponding points is: (i) choose three points from the ‘scene’ and ‘model’ point sets respectively; (ii) make a enumeration of the combination of these three points, estimate a possible transformation and finding if it could produce reasonable mapping; (iii) repeat the previous steps until a transformation that maximizes the congruent subset is found. It can be found that this procedure has two loops for 3-point combination, which requires $A_{N}^{3}A_{M}^{3}$ operations in average, resulting in an $O(M^{3}N^{3})$ complexity.). However, such complexity is prohibitively high considering the practically large number of fiducial markers and the number of micrographs.

#### 2.2.1 Local constrained 4-point invariant feature

Here, we propose a novel strategy, which combines the 4-point affine invariant feature (Aiger et al., 2008; Koenderink and van Doorn, 1991) with local constraints and invariant area ratio, to reduce the parameter searching space.

**4-point affine invariant feature:** For a point set $P=\{p_{a},p_{b},p_{c},p_{d}\}$ in which line $l_{ab}=p_{a}+\alpha (p_{b}-p_{a})$ intersect with line $l_{cd}=p_{c}+\beta (p_{d}-p_{c})$ at point $p_{e}$, the ratios $r_{1}=\frac{\left||p_{a}-p_{e}|\right|}{\left||p_{a}-p_{b}|\right|}$ and $r_{2}=\frac{\left||p_{c}-p_{e}|\right|}{\left||p_{c}-p_{d}|\right|}$ are preserved under any affine transformation. The 4-point set P along with the ratios *r*_1_ and *r*_2_ compose the 4-point invariant feature (A brief proof is provided in Supplementary S1.1).

Figure 1C and D demonstrate an example of the 4-point invariant feature, where Figure 1C shows the fiducial markers of the low tilt micrograph within the blue rectangle of Figure 1B, and D shows the correspondences of the high tilt micrograph within the red rectangle. By considering the intersections, point set {a,b,c,d} forms a 4-point invariant feature in Figure 1C and point set {a′,b′,c′,d′} forms the congruent invariant feature in Figure 1D. These two 4-point subsets could be fitted into each other within a suitable transformation (Fig. 1E).

**Local geometric constraint and numerical stability:** The fast inquiry of a 4-point invariant feature is still a problem for large point sets (Aiger *et al.*, 2008). Various local geometric constraints have been proposed to reduce the complexity, within which the nearest neighbor constraint is the most popular one (Amat *et al.*, 2008; Hauer et al., 2013; Vilas et al., 2016). Though the nearest neighbor constraint is able to prune the topology and reduce the complexity, the nearest neighbor itself, however, can be easily corrupted by outliers and distance changes. Figure 1C and D shows such an example, in which a 3-neighbors local linear embedding (LLE) system is built for point *a*. Unfortunately, affected by the missed detection of g′, we get points *g*, *d* and *h* as the 3-neighbors for point *a* in Figure 1C but points d′, h′ and c′ as the 3-neighbors for its correspondence in Figure 1D.

Here, we define lines $l_{ab}$ and $l_{cd}$ as the *diagonal* of an invariant feature P, and propose to extract the 4-point invariant feature within the length of diagonal constraints instead of the nearest neighbor geometry:

1. Build a nearest neighbor search tree (Bentley, 1975) to get the set of the distance $\left\{l_{k}\right\}$ between each point in a point set X with its nearest neighbor.
2. Get the average *l_avg_* and standard deviation *l_sdv_* of $\left\{l_{k}\right\}$.
3. Define a diagonal length for a 4-point invariant feature to be no more than $l_{\max}=3\sqrt{2}l_{\mathrm{avg}}+l_{\mathrm{sdv}}$.
4. Get all the possible point pairs within the point set X, and only select the point pairs with length no more than *l_max_* to produce the 4-point invariant feature.

By doing this, we have limited the extraction of 4-point invariant feature within a local constrained area (if the distribution of fiducial markers is approximately even, we will get all the 4-point invariant features within about 10-neighbors).

We further exclude the 4-point invariant feature with too small diagonal lengths: if the localization error for a fiducial marker is 5 pixels, a 4-point invariant feature with a diagonal length of 20 pixels will suffer from 5/20=25% inaccuracy in the invariant ratio, while a 4-point invariant feature with a diagonal length of 200 pixels will only suffer from 5/200 = 2.5% inaccuracy. The minimum diagonal length *l_min_* is related to the numerical stability of a given coordinate system, which depends on the nanoscale of fiducial markers and the pixel size of the micrograph. Here, we call this local geometry constraint the *min&max rule* for the 4-point invariant feature.

**Fig. 2.**
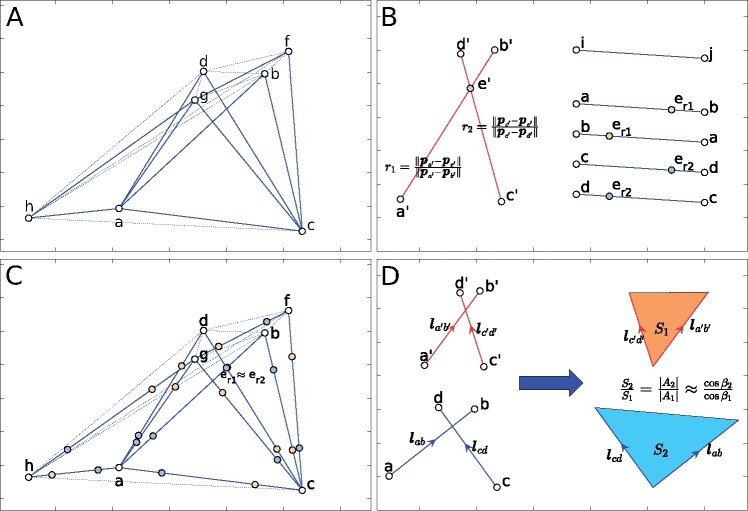
Fast inquiry and verification for a certain 4-point invariant feature. Given the minimum and maximum limitation of 4-point feature’s diagonal, a pruned virtual topology could be built on the ‘scene’ point set and the inquiry cost will be reduced when a 4-point feature of the ‘model’ point set comes. (**A**) A pruned virtual topology generated from the point set shown in Figure 1C, with the setting of minimal diagonal length *l_min_* = *l_gb_* and maximal length *l_max_* = *l_hg_*. (**B**) A 4-point feature chosen from the point set shown in Figure 1D, with the corresponding invariant ratio *r*_1_ and *r*_2_. (**C**) The inquiry of a consistent 4-point feature on the ‘scene’ point set. For each pair of points {*i*, *j*}, two kinds of possible intersection ($e_{r_{1}}$ and $e_{r_{2}}$) are calculated. A conflict of $e_{r_{1}}$ and $e_{r_{2}}$ indicates a possible candidate of the corresponding 4-point feature. (**D**) The area ratio constraint underlies the two corresponding 4-point features

Figure 2A shows an example of the ‘min&max rule’, where we set *l_min_* = *l_gb_* and *l_max_* = *l_hg_* for the convenience of demonstration. Here, for the points selected in Figure 1C, we have calculated all the possible point pairs $\left\{l_{p_{i}p_{j}}|p_{i},p_{j}\in X\right\}$. Then, the point pairs with $||l_{p_{i}p_{j}}||>l_{\max}$ or $||l_{p_{i}p_{j}}||<l_{\min}$ are discarded. Consequently, a pruned virtual topology is built based on the remaining point pairs. As shown in Figure 2A, about half of the point pairs that violate the min&max rule have been pruned from the topology, being plotted in dash lines. Furthermore, because of the min&max rule, there are few connections between the points depicted in Figure 2A and the other points outside the selected region, which limits the combination of the geometric features within a local area.

#### 2.2.2 Fast inquiry and verification of invariant feature

We could extract a ‘query’ subset from the ‘model’ point set Y and find its congruent subset in the ‘scene’ point set X. If we obtain an affine transformation from the inquiry that covers enough points after being applied to the ‘model’ point set, we may have found the correct initial transformation.

**Partial inquiry for a certain feature:** For a 4-point invariant feature P∈Y with invariant ratios *r*_1_ and *r*_2_, we try to check its correspondence in X based on the supposed position of its intersection *e*.

If a point pair $l_{ij}$ corresponds to one of P’s diagonals, it has

(2) $$\{\begin{array}{c} p_{e_{r_{1}(ij)}}=p_{i}+r_{1}(p_{j}-p_{i})\;\text{or}\;p_{e_{r_{1}(\text{ji})}}=p_{j}{+r}_{1}(p_{i}-p_{j}),\\ p_{e_{r_{2}(ij)}}=p_{i}+r_{2}(p_{j}-p_{i})\;\text{or}\;p_{e_{r_{2}(\text{ji})}}=p_{j}{+r}_{2}(p_{i}-p_{j}), \end{array}$$

where $e_{r_{1}(ij)},e_{r_{1}(ji)}$ represent the two types of intersection $e_{r_{1}}$ defined by *r*_1_, and $e_{r_{2}(ij)},e_{r_{2}(ji)}$ represent the two types of intersection $e_{r_{2}}$ defined by *r*_2_ (an illustration is shown in Fig. 2B). Consequently, to find P’s corresponding 4-point feature in X, we may build a nearest search tree of $\left\{e_{r_{2}}\right\}$ and search $e_{r_{1}}$ on the nearest search tree.

Figure 2C shows the detailed positions of the candidates $e_{r_{1}}$ and $e_{r_{2}}$ on the virtual topology, and how a candidate $e_{r_{1}}$ conflicts with a candidate $e_{r_{2}}$ (to make the figure clear, only the first type of $e_{r_{1}}$ and $e_{r_{2}}$ is depicted). Once a conflict of the query is detected, the corresponding subset will be selected as a candidate correspondence to the inquiry subset.

It can be found that the partial inquiry procedure will take $O(N^{2})$ time complexity even without the constraint of local geometry, which is much faster than the $O(N^{3})$ complexity method used by the 3-point combination in a baseline algorithm.

**Fast consistency verification:** Given a query subset as the 4-point invariant feature, multiple candidate correspondences may exist in the ‘scene’ point set. To retrieve the invariant feature that exactly corresponds to the query, the affine transformation should be estimated and tested on the entire point set, which is still time-consuming. Here, we introduce another affine constraint, the constraint of the area ratio, to simplify the verification:

Lemma Given a micrograph with tilt angle *β*_1_ and another one with tilt angle *β*_2_, the area of the corresponding plane shapes on these two micrographs have an approximate ratio of $\text{cos}\;\beta_{1}/\;\text{cos}\;\beta_{2}$.

The interested readers may refer to Supplementary S1.2 for the proof of the lemma. Figure 2D shows how the area ratio constraint is applied to the 4-point invariant feature. Here, it should be noted that the area ratio constraint is rotation and translation invariant. A relaxation parameter *ξ* is further defined. Given a candidate correspondence P′ of the 4-point invariant subset P, P′ is accepted if and only if $(1-\xi )\frac{\;\text{cos}\;\beta_{1}}{\;\text{cos}\;\beta_{2}}\;⩽\;\frac{S_{P}}{S_{P\prime }}\;⩽\;(1+\xi )\frac{\;\text{cos}\;\beta_{1}}{\;\text{cos}\;\beta_{2}}$.

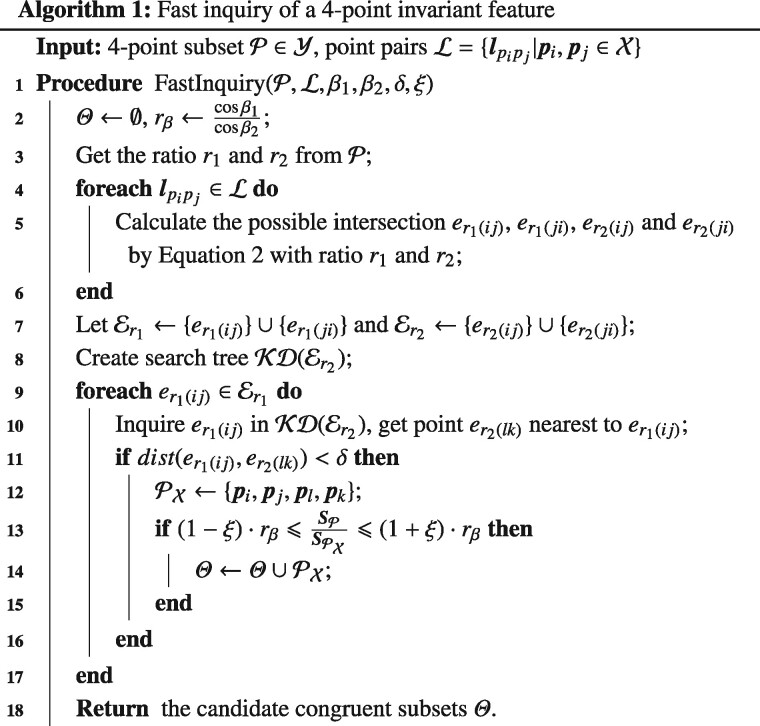

**Time complexity of the algorithm:**  Algorithm 1 summaries the proposed fast inquiry and verification algorithm, where the input is a 4-point invariant feature P extracted from the moving ‘model’ point set Y, the pruned point pairs ℓ extracted from the moving ‘scene’ point set X, the value of the two tilt angles *β*_1_, *β*_2_ and two parameters *δ*, *ξ* used to define the inquiry accuracy. $E_{r_{1}}$ and $E_{r_{2}}$ are the point sets generated from ℓ with ratios *r*_1_ and *r*_2_, and Θ is the candidate 4-point subset that correspond to P. KD(·) is the operation to build a k-d tree served for the nearest neighbor search (Bentley, 1975) and dist(·) is the operation to calculate the Euclidean distance between two points.

Because point pairs ℓ have been pruned by the min&max rule, the cardinality *K* of ℓ should be $\ll N^{2}$ (*N* is the cardinality of X). Then, the calculation of possible intersections has *O*(*K*) time complexity, building the nearest search tree has O(K log ⁡(K)) time complexity, and the query of the 4-point invariant subsets that correspond to P takes O(K log ⁡(K)) time complexity (O(log ⁡(K)) for each query and *O*(*K*) for the number of tries). Therefore, the total complexity of the algorithm is O(K+K· log ⁡(K)), which is far smaller than $O(N^{2}\;\text{log}\;(N))$.

#### 2.2.3 Robust estimation of the affine transformation

Considering the effect of noise and the missed detection of fiducial markers, multiple trials of congruent subset inquiry is necessary. Here, we adopt a random sample consensus (RANSAC) procedure (Fischler and Bolles, 1981) to robustly estimate the affine transformation between the two point sets.

Based on the min&max rule, the sets of range limited point pairs $\ell_{X}$ and $\ell_{Y}$ are generated from X and Y, respectively. Then, the following operations are carried out:

1. Compose a random 4-point set P by the point pairs within Y, and get its candidate congruent subsets Θ in X by the fast inquiry algorithm.
2. For each congruent subset $P_{X}$ within Θ, calculate the possible transform T(·;A,t) by the least square estimation. Apply the transform to Y and count the number of points in T(Y) that are close enough (congruent) to the points in X.
3. If the number of congruent points between T(Y) and X is larger than the current maximal record *c_max_*, save the current value as *c_max_* and update the termination condition *I*.
4. Repeat Steps 1 ∼4 until termination.

Algorithm 2 elaborates the details of the algorithm, where the algorithm accepts the point sets X, Y and the related tilt angles *β*_1_, *β*_2_ as input, the distance threshold *d*, the area ratio relaxation *ξ* as parameters, and outputs the estimated transformation T(·;A,t). The distance threshold *d* can be set according to the value of fiducial marker diameter and the relaxation *ξ* can be estimated based on the experiments of mechanical instability. Generally, *d* is set to λ·D, where *D* is the fiducial marker diameter and 0.5<λ<0.8. Because the fiducial markers are sparsely distributed on the specimen, the value of *λ* is not essential to the system. In the algorithm, we denote the operation to count the corresponding points between X and Y under distance threshold *d* as (X,Y,d). Because the transformation of Y needs a sequence of matrix multiplication, a further optimization is to randomize the verification of congruent points, by fast verifying a constant number of random points first and then the whole dataset. The maximum iteration is initialized and updated according to $I=\text{log}(1-p_{s})/\;\text{log}(1-p_{g}^{k})$, where *p_s_* is the required success probability, *p_g_* is the percentage of traceable fiducial markers that appear in both X and Y, and *k* is set to 4 as the inquiry of 4-point invariant subset.

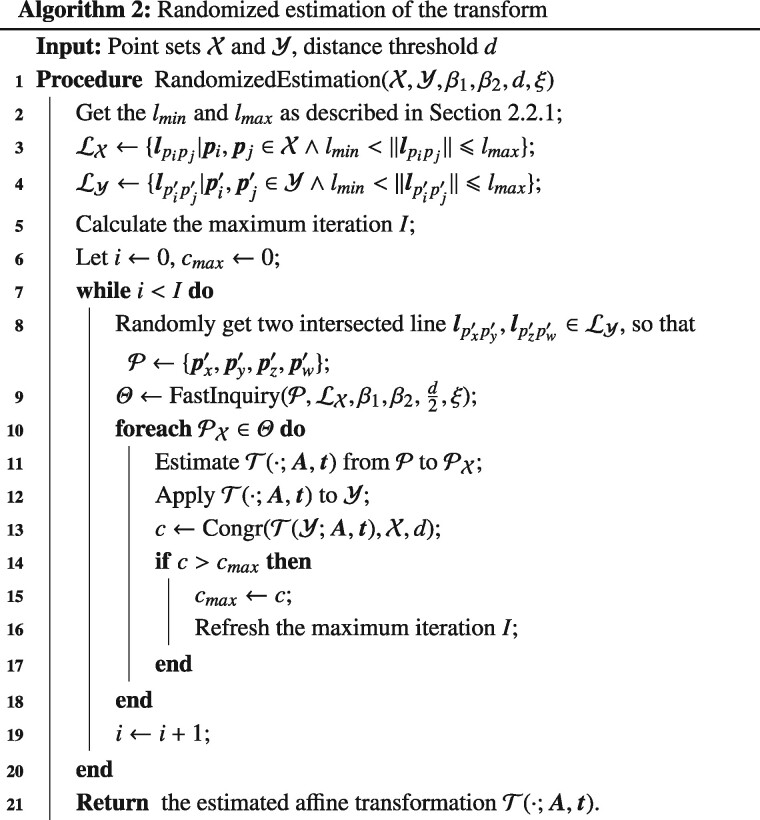

### 2.3 Local correspondence refinement

By considering the local geometric constraint, the optimized randomized algorithm is able to produce a fast and robust estimation of the initial transformation. However, the distortion caused by sample deformation or beam-induced motion is non-negligible (Fernandez *et al.*, 2019; Lawrence *et al.*, 2006; Zheng *et al.*, 2017), which may corrupt the affine relationship of two micrographs within a local area and lead to spurious correspondence.

Here, we first correct the large deviation and outliers in Y by the affine transformation T(·;A,t), and then feed the transformed ‘model’ point set and ‘scene’ point set to the algorithm for the second stage (for the concision of text, we still denote the corrected point set T(Y;A,t) by Y in the following discussion.).

#### 2.3.1 GMM interpolation for the scattered points

For the efficiency and accuracy, the data interpolation and transformation refinement are carried out by the non-rigid coherent point drift, based on the Gaussian mixture model (GMM) (Jian and Vemuri, 2011; Myronenko and Song, 2010).

Given the fixed ‘scene’ point set $X={\left\{x_{n}\right\}}_{n=1,\ldots ,N}$ and the moving ‘model’ point set $Y={\left\{y_{m}\right\}}_{m=1,\ldots ,M}$, the probability that a point x∈X is corresponding to a point y∈Y can be described by an isotropic Gaussian function:

(3) $$p(x|y)=\frac{1}{2\pi \sigma^{2}}\text{exp}\;\left(-\frac{||x-y|{|}^{2}}{2\sigma^{2}}\right),$$

where *σ* is a parameter to describe the instability of the system. Similarly, the probability that the point ***x*** belongs to the point set Y can be defined as $p(x|Y)=\sum_{m=1}^{M}P(m)p(x|y_{m})$, where *P*(*m*) is the prior of ***x*** under the condition of the *m*th point $y_{m}$.

Considering a drift transform T(·;v) applied to the model for distortion correction, and assuming the point ***x*** either belongs to outliers with *w* probability or sampled from the point set Y with a uniform distribution, the GMM probability density function can be defined as:

(4) $$\begin{array}{c} p(x|v_{m})=w\frac{1}{N}+(1-w)\sum_{m=1}^{M}\frac{1}{M}p(x|y_{m}),\\ =w\frac{1}{N}+(1-w)\sum_{m=1}^{M}\frac{1}{M}\frac{1}{2\pi \sigma^{2}}e^{-\frac{||x-T(y_{m};v_{m})|{|}^{2}}{2\sigma^{2}}}, \end{array}$$

where $v_{m}$ is the drift corresponding to the *m*th point $y_{m}$. Our aim is to find such a transformation T(·;v) and parameter *σ* applied to all the points y∈Y so that the negative log-likelihood from Y to X is minimized:

(5) $$E(T(\cdot ),\sigma^{2})=-\sum_{n=1}^{N}\;\text{log}\;\sum_{m=1}^{M+1}P(m)p(x|y_{m}),$$

where *P*(*m*) is the reweighted i.d.d prior and $p(x|y_{M+1})=\frac{w}{N}$ presents the probability of outliers.

#### 2.3.2 Transform parameter optimization

A negative log-likelihood objective function could be effectively solved by the expectation-maximization (E-M) optimization, where the algorithm iterates between the E-step and the M-step until convergence.

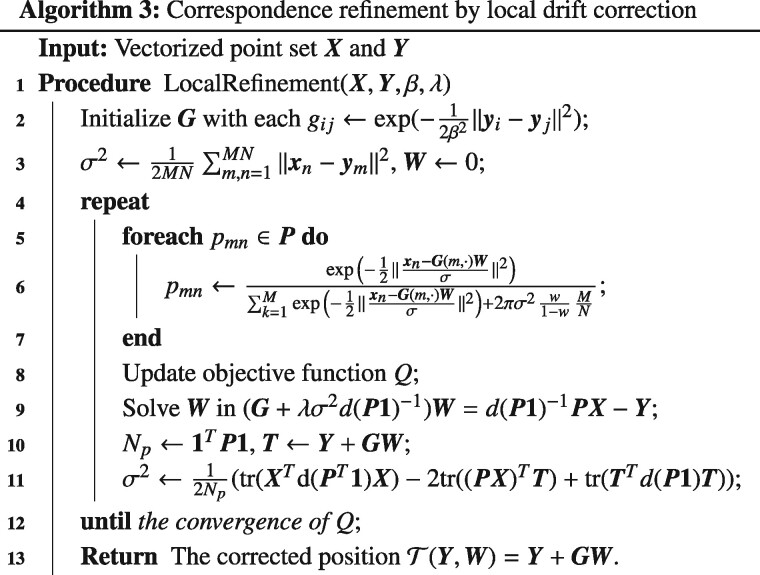

With Jensen’s inequality (Jensen, 1906; Redner and Walker, 1984), the negative log-likelihood defined in Eq. 5 is upper bounded by the following function in each iteration:

(6) $$Q=-\sum_{n=1}^{N}\sum_{m=1}^{M+1}p(m|x_{n})\;\text{log}\;\left(P^{\mathrm{new}}(m)p^{\mathrm{new}}(x_{n}|y_{m})\right),$$

where $p(m|x_{n})=P(m)p(x_{n}|y_{m})/p(x_{n})$ is the probability that $y_{m}$ corresponds to $x_{n}$, the ‘old’ superscript indicates that a parameter is guessed in the E-step and the ‘new’ superscript indicates that a parameter is optimized from the negative log-likelihood function in the M-step.

**E-step:** By ignoring the constants independent of ***v*** and *σ*, we rewrite Eq. 6 as:

(7) $$Q(v,\sigma^{2})=\frac{1}{2\sigma^{2}}\sum_{n=1}^{N}\sum_{m=1}^{M}p(m|x_{n})||x_{n}-T(y_{m};v_{m})|{|}^{2}+N_{p}\;\text{log}\;\sigma^{2},$$

where $N_{p}=\sum_{n=1}^{N}\sum_{m=1}^{M}p(m|x_{n})\;⩽\;N$ (with *N* = *N_p_* only if *w *=* *0), and $p(m|x_{n})$ denotes the posterior probabilities of GMM components calculated using the previous parameter values:

(8) $$p(m|x_{n})=\frac{\;\text{exp}\;\left(-\frac{1}{2}||\frac{x_{n}-T(y_{m},v_{m})}{\sigma }|{|}^{2}\right)}{\sum_{k=1}^{M}\;\text{exp}\;\left(-\frac{1}{2}||\frac{x_{n}-T(y_{k},v_{m})}{\sigma }|{|}^{2}\right)+2\pi \sigma^{2}\frac{w}{1-w}\frac{M}{N}}.$$

**M-step**: Here we model the transform T(·;v) to correct the local distortion but not global affine transformation. For the convenience of further discussion, here we introduce the following notations:

$X_{N\times 2}={\left(x_{1}\cdots x_{N}\right)}^{T}$ —matrix presentation of the point set X;

$Y_{M\times 2}={\left(y_{1}\cdots y_{M}\right)}^{T}$ —matrix presentation of the point set Y;
**1**—the column vector of all ones;
4. d(a)—the diagonal matrix formed from vector ***a***;
***P***—the matrix that is composed by $p_{mn}=p(m|x_{n})$.

Based on the Tikhonov regularization framework (Chen and Haykin, 2002), a non-rigid parameterization for T(·;v) is adapted to minimize the objective function *Q*:

(9) T(Y;v)=Y+v(Y),

where v(·) is the expected drift. A regularization term $\frac{\lambda }{2}\phi (v)$ is added to Equation 7 to enforce the smoothness and compensate for the drift, and *λ* is a trade-off parameter.

If we model the regularization function $\phi (v)=||\text{Lv}|{|}^{2}$ within a Kernel Hilbert Space (RKHS) (Chen and Haykin, 2002; Myronenko and Song, 2010), the negative log-likelihood function in Equation 7 will be defined as:

(10) $$\begin{array}{c} Q(v,\sigma^{2})=\frac{1}{2\sigma^{2}}\sum_{n=1}^{N}\sum_{m=1}^{M}p(m|x_{n})||x_{n}-(y_{m}+v(y_{m}))|{|}^{2}\\ +N_{p}\;\text{log}\;\sigma^{2}+\frac{\lambda }{2}||\text{Lv}|{|}^{2}. \end{array}$$

To minimize Equation 10, we should find a function v(·) for all the elements $y_{m}$ of ***Y*** under the Euler-Lagrange differential equation:

(11) $$\frac{1}{\sigma^{2}\lambda }\sum_{n=1}^{N}\sum_{m=1}^{M}p(m|x_{n})(x_{n}-(y_{m}+v(y_{m})))\Delta y_{m}=\hat{\text{L}}\text{Lv}(\Delta y_{m}),$$

where $\hat{\text{L}}$ is the adjoint operator to ***L***. By rewriting the equation and using an integral of a Green’s function $g(x,y)=e^{-\frac{1}{2}||\frac{x-y}{\beta }|{|}^{2}}$ instead of the self-adjoint operator, the solution v(·) of such a partial differential equation has the form of

(12) $$\begin{array}{c} v(z)=\frac{1}{\sigma^{2}\lambda }\sum_{n=1}^{N}\sum_{m=1}^{M}p(m|x_{n})(x_{n}-(y_{m}+v(y_{m})))g(z,y_{m})\\ =\sum_{m=1}^{M}w_{m}g(z,y_{m}), \end{array}$$

where $w_{m}=\frac{1}{\sigma^{2}\lambda }\sum_{n=1}^{N}p(m|x_{n})(x_{n}-(y_{m}+v(y_{m})))$. To further get the values of v(·), we could solve each $w_{m}$ first by evaluating Eq.12 at $y_{m}$:

(13) $$(G+\lambda \sigma^{2}d{\left(\text{P1}\right)}^{-1})W=d{\left(\text{P1}\right)}^{-1}\text{PX}-Y,$$

where $W={\left(w_{1},\ldots ,w_{M}\right)}^{T}$, ***G*** is an *M *×* M* kernel matrix with elements $g_{ij}=g(y_{i},y_{j})$, and $d{\left(\cdot \right)}^{-1}$ is the inverse diagonal operation.

Consequently, the transform T=T(Y;W)=Y+GW. By substituting ***T*** back into *Q* and solving the partial derivation, $\sigma^{2}$ is updated according to the result of $\frac{\partial Q}{\partial \sigma^{2}}$ as

(14) $$\sigma^{2}=\frac{1}{2N_{p}}(\text{tr}(X^{T}d(P^{T}1)X)-2\text{tr}({\left(\text{PX}\right)}^{T}T)+\text{tr}(T^{T}d(\text{P1})T)).$$

The overall iterative optimization for the GMM-based drift correction is summarized in Algorithm 3. After the update of the positions by T(Y,W), we could recalculate the correspondence between point sets Y and X under a given distance threshold *d*. With the initial transformation inherited from the first stage, Algorithm 3 is able to quickly converge. At the same time, because the initial transformation is very close to the global optimum, Algorithm 3 will converge to the global optimum in a high probability.

## 3 Experiments and results

### 3.1 Datasets

Six real-world datasets are used to evaluate the proposed method. The first dataset is a tilt series that has been used in the previous studies, provided by the Institute of Biophysics, Chinese Academy of Sciences (Han *et al.*, 2018). The remaining five datasets are downloaded from the Caltech ETDB (Ortega et al., 2019).

**Fig. 3.**
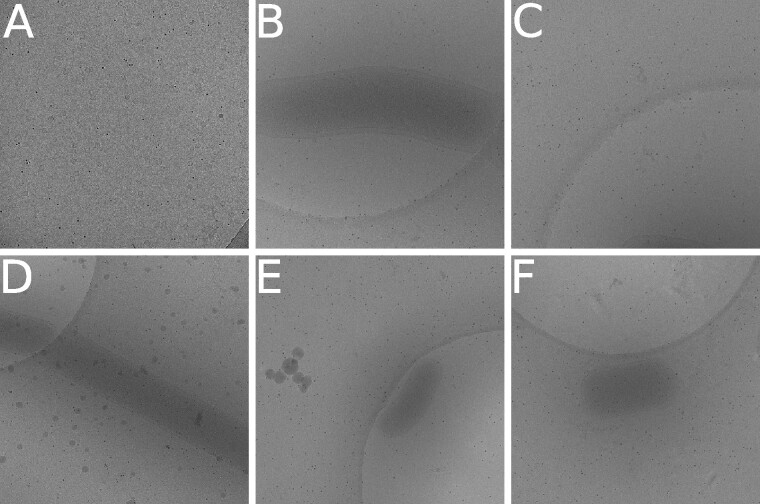
Illustration of the test datasets. (**A**) Hemocyanin, (**B**) Vibrio1, (**C**) Vibrio2, (**D**) Nitrosop1, (**E**) Nitrosop2 and (**F**) Nitrosop3. Limited by the space, only the small thumbnails of the $0^{{}^{\circ}}$ micrographs are shown here. Please refer to Supplementary S2.1 for detailed information

The first dataset, Hemocyanin, is a tilt series of vitrified keyhole limpet hemocyanin solution (Fig. 3A). It is a cryo-ET dataset with about 100 ∼150 fiducial markers embedded in. The tilt series were collected by FEI Titan Krios (300 kV) with a Gatan US4000 camera. The total dose used during data collection was around 8000 e/nm^2^. There are 95 images with the tilt ranging from $-70^{{}^{\circ}}$ to $70^{{}^{\circ}}$ at $1^{{}^{\circ}}∼2^{{}^{\circ}}$ intervals (2K×2K pixels with 0.4 nm/px).

The second and third datasets, Vibrio1-2 (Vibrio1 is downloaded from https://bit.ly/35HJoWS; Vibrio2 is downloaded from https://bit.ly/3kGvl8l.), are two cryo-ET datasets of isolated Vibrio cholerae cells (Fig. 3B and C). Vibrio1 and Vibrio2 have about 150 ∼200 and 200 ∼250 fiducial markers embedded in the specimens, respectively. Both of the tilt series were collected by FEI Tecnai Polara (F30) (300 kV) with a Gatan K2 camera, operated at $145\text{eV}/{Å}^{2}$ dosage. There are 121 images with the tilt ranging from $-60^{{}^{\circ}}$ to $60^{{}^{\circ}}$ at $1^{{}^{\circ}}$ interval (4K×4K pixels with 0.4 nm/px).

The fourth to sixth datasets, Nitrosop1-3 (Nitrosop1 is downloaded from https://bit.ly/3pCgsHL; Nitrosop2 is downloaded from https://bit.ly/36L2zyq; Nitrosop3 is downloaded from https://bit.ly/3kF1QUb.), are three cryo-ET datasets of isolated Nitrosopumilus maritimus cells (Fig. 3D–F). Nitrosop1 has about 250 ∼300 fiducial markers embedded in the specimen, while both Nitrosop2 and Nitrosop3 have about 400 ∼500 fiducial markers. The Nitrosop1-3 were collected by FEI Tecnai Polara (F30) (300 kV) with a Gatan K2 camera. The Nitrosop1 was operated at $150\text{eV}/{Å}^{2}$ dosage with –10 *μ*m defocus; the Nitrosop2 and Nitrosop3 were operated at $180\text{eV}/{Å}^{2}$ dosage. There are 121 images for Nitrosop1 with the tilt ranging from $-60^{{}^{\circ}}$ to $60^{{}^{\circ}}$ at $1^{{}^{\circ}}$ interval (4K×4K pixels with 0.64 nm/px), and 111 images for both Nitrosop2 and Nitrosop3 with the tilt angles ranging from $-55^{{}^{\circ}}$ to $55^{{}^{\circ}}$ at $1^{{}^{\circ}}$ interval (4K×4K pixels with 0.49 nm/px).

As our focus is on the determination of fiducial marker correspondence, all the fiducial markers on the micrographs have been detected in advance. Here, we used the sampling and classification algorithm proposed in *markerauto* (Han *et al.*, 2015) to automatically and exhaustively detect the fiducial markers. Nevertheless, other sophisticated techniques can also be used to provide precise fiducial marker positions.

### 3.2 Results

#### 3.2.1 Robustness of the algorithm under various conditions

The robustness is very important for a fully automatic fiducial marker correspondence method. Here, we first test the robustness of the proposed method on the datasets with different micrograph pairs.

**Fig. 4.**
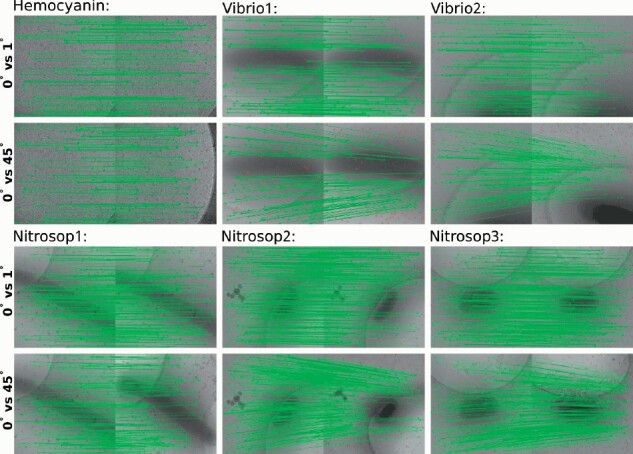
A snapshot of the fiducial marker correspondence determined by the two-stage algorithm on micrographs with different tilt angle intervals (for the demonstrated micrograph pairs, the left is with $0^{{}^{\circ}}$ tilt angle and the right is with $1^{{}^{\circ}}$ or $45^{{}^{\circ}}$ tilt angle, respectively). Please refer to Supplementary S2.2 for detailed information

For each dataset, the tracking of fiducial markers on the micrographs with tilt angle intervals increasing from $1^{{}^{\circ}}$ to $45^{{}^{\circ}}$ is carried out by the two-stage algorithm. Figure 4 demonstrates a snapshot of the tracking results, in which the experimental results on micrographs with tilt angles of $0^{{}^{\circ}}$ and $1^{{}^{\circ}},\;0^{{}^{\circ}}$ and $45^{{}^{\circ}}$ are selected for illustration. It should be noted that, from the dataset Hemocyanin to Nitrosop3, the number of fiducial markers has increased from one hundred to more than five hundred. Nevertheless, for all the datasets with various tilt angle intervals, our algorithm successfully finds the correct correspondence.

**Fig. 5.**
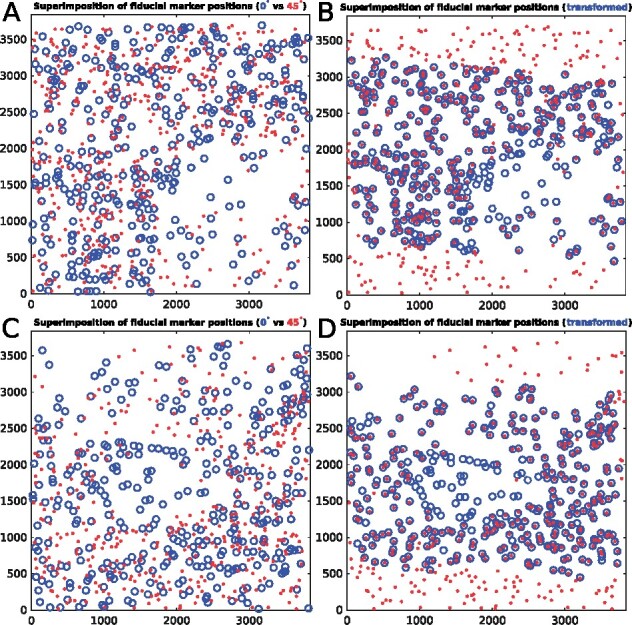
Superimposition of fiducial marker positions from the $0^{{}^{\circ}}$ and $45^{{}^{\circ}}$ tilted micrograph before (the left) and after (the right) applying transformation produced by the two-stage algorithm. (**A**) and (**B**) fiducial marker positions extracted from the Nitrosop2 dataset. (**C**) and (**D**) fiducial marker positions extracted from the Nitrosop3 dataset

Particularly, the proposed method could handle the conditions with numerous fiducial markers and a mass of outliers well. Figure 5 illustrates the superimposed fiducial marker positions of the Nitrosop2 and Nitrosop3 datasets that are extracted from the micrographs with $0^{{}^{\circ}}$ and $45^{{}^{\circ}}$ tilt angles (labeled by blue ‘circle’ and red ‘dot’, respectively). The two-stage algorithm accepts the raw fiducial marker positions shown in Figure 5A and C as input and outputs the transformed fiducial marker positions as shown in Figure 5B and D. There are 394 and 366 detected fiducial markers on the $0^{{}^{\circ}}$ tilted micrographs of the Nitrosop2 and Nitrosop3 datasets, and 513 and 452 detected fiducial markers on the $45^{{}^{\circ}}$ tilted micrographs of the Nitrosop2 and Nitrosop3 datasets, respectively. As shown in Figure 5A and C, the fiducial markers from the $0^{{}^{\circ}}$ and $45^{{}^{\circ}}$ tilted micrographs are difficult to be directly corresponded together. Especially, for the micrographs with high tilt angles, the blurring and blocking of fiducial markers cause a mass of missed detection. In Figure 5B and D, we use the green ellipses to indicate the missed detection of fiducial markers caused by the increasing dark shadows and use the red ellipses to indicate the introducing of outliers with the extension of the field of view. In total, there are more than one hundred fiducial markers appearing as outliers and hampering the estimation of transformation. However, after the execution of the algorithm, the proposed method has generated high-quality fiducial marker correspondence.

**Fig. 6.**
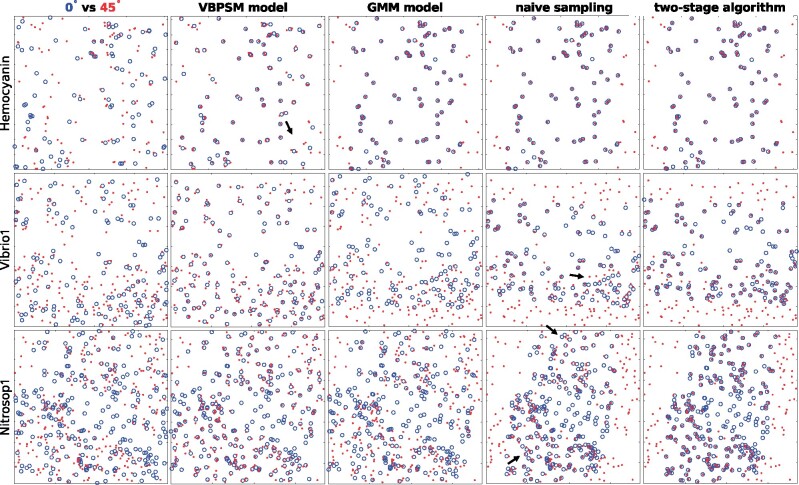
Performance comparison between different fiducial marker tracking methods. The 1st column illustrates the superimposition of the raw fiducial marker positions, where the blue ‘circle’ and red ‘dot’ denote the fiducial markers extracted from the $0^{{}^{\circ}}$ and $45^{{}^{\circ}}$ tilted micrographs, respectively. The 2nd, 3rd, 4th and 5th columns illustrate the transformed fiducial marker positions of the $0^{{}^{\circ}}$ tilted micrograph solved by the VBPSM model, the GMM model, naive sampling and the two-stage algorithm, respectively. Due to space limitation, only the experimental results of the Hemocyanin, Vibrio1 and Nitrosop1 datasets are shown here. Please refer to Supplementary Figure S14 for the results of the other datasets

The proposed method is then compared with the state-of-the-art methods, including the probabilistic graphical model for robust point set registration (Qu et al., 2017), the GMM-based fast fiducial marker tracking model (Han *et al.*, 2018) and the affine transform-based naive sampling method (Han *et al.*, 2015), which are referred to as ‘VBPSM model’, ‘GMM model’ and ‘naive sampling’, respectively [Because RAPTOR (Amat *et al.*, 2008) needs a pre-alignment of the tilt series and cannot be applied on micrographs with large tilt intervals (for example, intervals $>15^{{}^{\circ}}$), we compare with the VBPSM model instead of RAPTOR, which uses a similar probabilistic graphical model as RAPTOR does. IMOD’s another fiducial marker correspondence solution, beadtrack script, is based on nearest neighbor search, which cannot solve the problem with large deviation or tilt angles. Thus, it is also not compared here.]. Figure 6 demonstrates the comparative results of these methods on the tilted micrographs with $0^{{}^{\circ}}$ and $45^{{}^{\circ}}$ tilt angles. To finish the task, the two-stage algorithm, the GMM model and the VBPSM model cost hundreds of milliseconds to several minutes for each dataset, whereas the naive sampling method costs tens of minutes.

As shown in the 1st column of Figure 6, the distributions of fiducial marker positions on the $0^{{}^{\circ}}$ and $45^{{}^{\circ}}$ tilted micrographs cannot be easily aligned. The Hemocyanin dataset has a relatively small number of fiducial markers and fewer outliers. Consequently, the correspondences are successfully determined by all the methods, though the VBPSM model has failed in several local areas. With the increase of the fiducial marker number and outliers, the VBPSM model and GMM model performed poorly on the Vibrio1 and Nitrosop1 datasets. Though the methods tried to maximize the correlation of the underlying geometric information depicted by the fiducial markers, the numerous outliers resulted in a trap of local optimum. On the contrary, the naive sampling and the two-stage algorithm produced reasonable results. However, as pointed out by the arrows in Figure 6, the results of naive sampling suffer from a severe non-uniform deviation, whereas the two-stage algorithm produces a tight fitting.

#### 3.2.2 Efficiency and effectiveness of the algorithm

When the tilt angle and the field of the view increase, an affine transformation is not able to accurately describe the potential correspondence related to two tilted micrographs. With the introducing of local drift refinement, the two-stage algorithm can easily solve the problem.

**Fig. 7.**
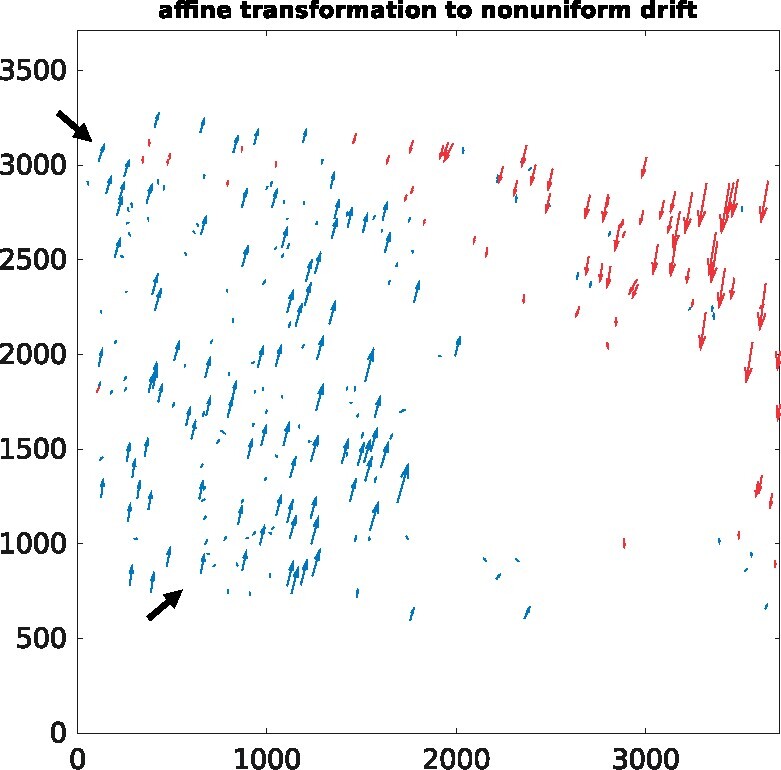
Demonstration of the non-uniform drift of the fiducial markers. The presented drift vectors are estimated from the $0^{{}^{\circ}}$ and $45^{{}^{\circ}}$ tilted micrographs of Nitrosop2 (Fig. 5A and B). Here, the drifts with different directions are marked by different colors. Please refer to Supplementary Figure S15 for the other datasets

Figure 7 shows the non-uniform drift of the fiducial markers solved from Figure 5A and B, in which the drift is estimated based on the initial affine transformation solved in the first stage. There are two drift directions shown in Figure 7, the drift upward (marked by blue) on the left and the drift downward (marked by red) on the right, which may be caused by a considerable global deformation of the sample. Furthermore, by analyzing the drift direction pointed out by the black arrows in Figure 7, an additional local non-uniform deformation can be noticed. The fiducial markers embedded nearby or on the close depths may have a similar drift, as shown in the right top of Figure 7. Generally, most of the fiducial markers are laid on the surface of the specimen, thus, the deviations of the fiducial markers on two micrographs will grow in a gradient way. If there are two neighbor fiducial markers with very close *x–y* distance but with quite different *z* distance (different heights) in the space, they may result in a very different drift magnitude, even though both markers appear next to each other in the projection image, which could be observed in Figure 7. However, with a correct partition of the fiducial markers and local drift correction, the two-stage algorithm can efficiently discover the underlying local drift.

In practice, for the tilt series alignment, we may track several neighbors of the micrographs and then compose the fiducial marker correspondences into the fiducial marker tracks. Here, for all the datasets, we matched the fiducial marker positions within 2-neighbors micrographs, i.e. the *n*th and (n+1)th, and *n*th and (n+2)th micrographs, by the GMM model, the naive sampling and the proposed algorithm. Within each matching operation, the ratio of the matched fiducial marker pairs to the total number of potential correspondence was calculated. We also tested RAPTOR on the datasets, based on the prealigned tilt series produced in IMOD. However, limited by the runtime and execution error, RAPTOR failed to run on the Nitrosop2 and Nitrosop3 datasets. All of the methods were run on a Fedora 25 system with 128 Gb memory and two E5-2667v4 (3.2 GHz) CPU.

Considering that the deformation usually happens in high tilt angles, we calculated the average detection ratio of micrograph pairs with tilt angle $\;⩾\;30^{{}^{\circ}}$ or $\;⩽-30^{{}^{\circ}}$. Table 1 summaries the averaged detection ratio for each method and dataset. As shown in Table 1, all the methods perform quite well on the Hemocyanin dataset, which has a relatively small field of view (2K×2K) and contains about one hundred fiducial markers only. However, for the other datasets which contain larger numbers of fiducial markers, the two-stage algorithm performs much better than the other methods. For the datasets of Nitrosop2 and Nitrosop3, the accuracy was increased from ∼94% of the GMM model and the naive sampling method to ∼99% of the two-stage algorithm. Here, the gain of accuracy mainly comes from the model flexibility introduced by the second stage of the algorithm. The experimental results demonstrate the effectiveness of the two-stage algorithm in fiducial marker correspondence for micrographs with wide field and numerous fiducial markers.

**Table 1.** The correspondence accuracy of the methods on high tilted micrographs

	RAPTOR	Naive sampling	GMM model	Two-stage algorithm
Hemocyanin	92.1%	97.5%	98.2%	99.2%
Vibrio1	84.3%	93.3%	95.4%	98.6%
Vibrio2	81.8%	94.3%	94.5%	99.1%
Nitrosop1	78.7%	93.1%	95.2%	98.7%
Nitrosop2	—	91.9%	94.6%	98.9%
Nitrosop3	—	92.6%	94.8%	99.2%

**Fig. 8.**
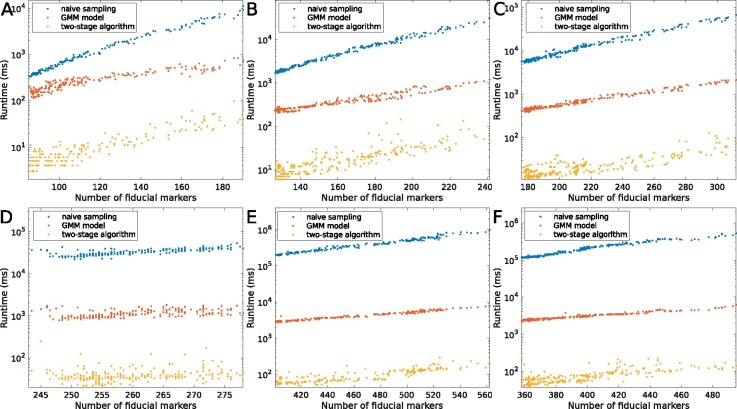
Runtime of the proposed two-stage algorithm (in yellow), the GMM-based fast tracking (in red) and the naive sampling (in blue) on (**A**) Hemocyanin, (**B**) Vibrio1, (**C**) Vibrio2, (**D**) Nitrosop1, (**E**) Nitrosop2 and (**F**) Nitrosop3. The *x*-axis represents the average number of fiducial markers and the y-axis represents the runtime (ms) in the log scale

The efficiency is also a critical factor when a new algorithm is applied to the real-world datasets. For each matching operation, the runtime of different methods against the number of fiducial markers is recorded. Figure 8 illustrates the corresponding runtime for each dataset, where the x-axis represents the average number of fiducial marker positions for each matching operation, and the y-axis represents the runtime (ms) in the log scale (integrated within IMOD, RAPTOR’s per image runtime is not available). Table 2 summaries the total runtime for each dataset with different methods.

**Table 2.** The total runtime of fiducial marker tracking for each tilt series

	RAPTOR	naive sampling	GMM model	two-stage algorithm
Hemocyanin	14.5 min	6.4 min	58.1 s	2.6 s
Vibrio1	2.40 h	24.5 min	1.6 min	5.0 s
Vibrio2	2.51 h	1.01 h	3.0 min	5.8 s
Nitrosop1	3.36 h	2.16 h	4.5 min	10.0 s
Nitrosop2	—	21.01 h	15.2 min	21.1 s
Nitrosop3	—	12.60 h	11.4 min	17.3 s

As shown in Figure 8A, all of the fiducial marker tracking methods have acceptable execution efficiency when there are not too many fiducial markers. However, with the increasing of the fiducial marker number, the runtime of RAPTOR and the naive sampling surge dramatically. As shown in Figure 8E and F, for a dataset with about five hundreds fiducial markers, the execution time of the naive sampling method has increased to ∼8 min per matching, reaching 21 and 12.6 h for the entire tilt series of Nitrosop2 and Nitrosop3, respectively. The GMM model has a relatively low cost compared to the naive sampling. Nevertheless, it still costs more than 15 and 10 min for the entire tilt series of Nitrosop2 and Nitrosop3, respectively. On the contrary, the average runtime of the two-stage algorithm has been controlled within 100 ms per micrograph matching for almost all the datasets. Specifically, the two-stage algorithm only costs 21.1 s and 17.3 s for the Nitrosop2 and Nitrosop3 datasets, which is 40× faster than the GMM-based fast-tracking method and 1000× faster than the native sampling method.

#### 3.2.3 Case study within the tilt series alignment workflow

Finally, we integrated the two-stage algorithm into the fully automatic alignment scheme (Han *et al.*, 2015) to further verify its application in an end-to-end workflow.

In the alignment scheme proposed by Han *et al.* (2015), the fiducial markers are firstly detected and refined. Then, with the refined fiducial marker positions, a fiducial marker correspondence algorithm is executed to guarantee the consistent matching of the fiducial marker positions from neighboring micrographs. When the required fiducial marker correspondences are obtained, a set of fiducial marker tracks will be generated and the projection parameters are able to be optimized with the configured tracks, to finally determine the geometric transformation of the micrographs in tilt series alignment. Here, the original marker correspondence algorithm will be replaced by our new two-stage algorithm, and the affine projection model is used in projection parameter optimization.

**Fig. 9.**
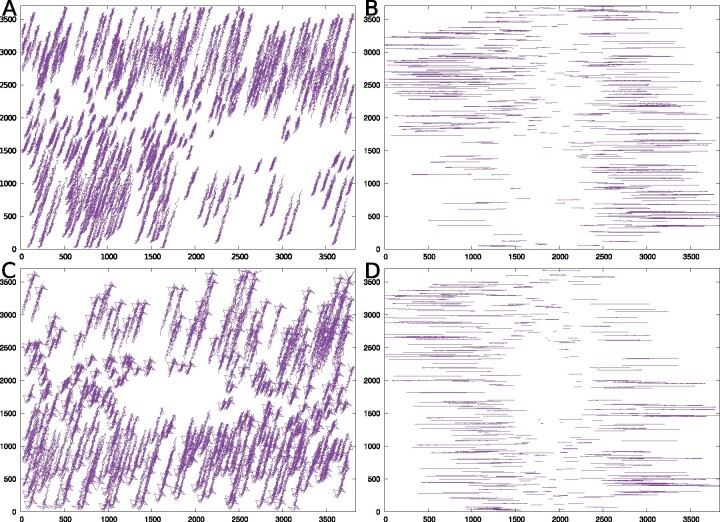
Illustration of tilt series alignment with the two-stage algorithm used for fiducial marker tracking. (**A**) Overlay of raw fiducial marker tracks (*x*–*y* coordinates) extracted from the Nitrosop2 dataset. (**B**) Overlay of the aligned fiducial marker tracks of the Nitrosop2 dataset. (**C**) Overlay of raw fiducial marker tracks extracted from the Nitrosop3 dataset. (**D**) Overlay of the aligned fiducial marker tracks of the Nitrosop3 dataset

Here, we demonstrate the alignment results of the Nitrosop2 and Nitrosop3 datasets as the case study. In the tilt series alignment, only the tracks with enough length are used for projection parameter estimation. For the Nitrosop2 dataset, the two-stage algorithm produced 338 fiducial marker tracks that cover at least 70% of the entire tilt series, with an average track length of 105.9 (Fig. 9A). After the optimization of projection parameters, these fiducial marker tracks resulted in a mean alignment residual of 0.69 pixels (Fig. 9B). For the Nitrosop3 dataset, the two-stage algorithm produced 302 fiducial marker tracks that cover at least 70% of the entire tilt series, with an average track length of 105.3 (Fig. 9C). After the optimization of projection parameters, these fiducial marker tracks resulted in a mean alignment residual of 0.47 pixels (Fig. 9D). Here, the difference in residual error between the two datasets may be caused by the serious sample deformation in the Nitrosop2 dataset (Fig. 7), which can be solved by a higher-order projection model (Fernandez *et al.*, 2018; Lawrence *et al.*, 2006). However, judging from the horizontal fiducial marker tracks after the correction of in-plane rotation and translation (Fig. 9B and D), the orthogonal projection is still able to produce a successful alignment, based on the fiducial marker tracks produced by the two-stage algorithm.

## 4 Conclusion and discussion

In this article, we proposed a novel two-stage algorithm for the fiducial marker correspondence in electron tomography. The aim of this work is to completely solve the fiducial marker tracking problem in a robust and ultrafast way. The algorithm combines both the robustness of the combinatorial search and the inherent flexibility of the probabilistic model, to further improve the accuracy of fiducial marker correspondence. Generally, the two-stage algorithm can solve the fiducial marker correspondence with arbitrary initial positions of the micrographs within just a few seconds.

Currently, there are more than ten thousand of the tilt series within the ETDB (Ortega *et al.*, 2019), and the database keeps exploding with the wide application of the ET technique, which raises the emergency demand for a well-designed, high-performance fully automatic software. The improved fiducial marker correspondence accuracy could be used to generate more complete fiducial marker tracks in a more efficient way. With the aid of a large number of well-tracked fiducial markers, a detailed study in projection model selection and validation is possible, especially on the datasets with a serious sample deformation. Cooperation with the world-famous groups (Fernandez *et al.*, 2018; Lawrence *et al.*, 2006) is expected to make follow-up research about robust large-scale bundle adjustment with non-linear projection model, which may further improve the accuracy of tilt series alignment. The related code of our method is also shared online, interested readers may utilize the code to build their own efficient and automatized software.

## Funding

This work was supported by the National Key Research and Development Program of China [2020YFA0712400], the National Natural Science Foundation of China [62072280, 11931008, 61771009], the King Abdullah University of Science and Technology (KAUST) Office of Sponsored Research (OSR) under Awards No. FCC/1/1976-17, FCC/1/1976-23, FCC/1/1976-26, URF/1/4098-01-01, URF/1/4352-01-01, URF/1/4379-01-01, REI/1/0018-01-01 and REI/1/4473-01-01.

*Conflict of Interest*: none declared.

## Supplementary Material

btaa1098_Supplementary_Data
