# Supplementary material for: Robust and ultrafast fiducial marker correspondence in electron tomography by a two-stage algorithm considering local constraints
Source: Bioinformatics. 2021 Jan 8;37(1):107–17. doi: 10.1093/bioinformatics/btaa1098 (PMC8694346; doi:10.1093/bioinformatics/btaa1098)
Supplement: btaa1098_Supplementary_Data [file btaa1098_supplementary_data.pdf]

Robust and ultrafast fiducial marker correspondence in electron tomography by a two-stage algorithm considering local constraints

Renmin Han[1,2], Guojun Li[1,*], Xin Gao[2,*]

[1]Research Center for Mathematics and Interdisciplinary Sciences, Shandong University, Qingdao 266237, PR China
[2]King Abdullah University of Science and Technology (KAUST), Computational Bioscience Research Center (CBRC), Computer, Electrical and Mathematical Sciences and Engineering (CEMSE) Division, Thuwal, 23955-6900, Saudi Arabia

All correspondence should be addressed to Xin Gao (xin.gao@kaust.edu.sa) or Guojun Li (guojunsdu@gmail.com).

S1 Methods

S1.1 Proof for 4-point invariant ratio

For a point set $\mathcal{P} = \{\boldsymbol{p}_a, \boldsymbol{p}_b, \boldsymbol{p}_c, \boldsymbol{p}_d\}$ in which line $\boldsymbol{l}_{ab} = \boldsymbol{p}_a + \alpha(\boldsymbol{p}_b - \boldsymbol{p}_a)$ intersect with line $\boldsymbol{l}_{cd} = \boldsymbol{p}_c + \beta(\boldsymbol{p}_d - \boldsymbol{p}_c)$ at point $\boldsymbol{p}_e$, the ratios $r_1 = \frac{\|\boldsymbol{p}_a - \boldsymbol{p}_e\|}{\|\boldsymbol{p}_a - \boldsymbol{p}_b\|}$ and $r_2 = \frac{\|\boldsymbol{p}_c - \boldsymbol{p}_e\|}{\|\boldsymbol{p}_c - \boldsymbol{p}_d\|}$ are preserved under any affine transformation. The 4-point set $\mathcal{P}$ along with the ratios r_1 and r_2 compose the 4-point invariant feature:

Brief proof: According to the premise, for the intersection $\boldsymbol{p}_e$, $\boldsymbol{p}_e = \boldsymbol{p}_a + r_1(\boldsymbol{p}_b - \boldsymbol{p}_a)$ and $\boldsymbol{p}_e = \boldsymbol{p}_c + r_2(\boldsymbol{p}_d - \boldsymbol{p}_c)$. Applying an arbitrary affine transformation $\mathcal{T}(\cdot; \boldsymbol{A}, \boldsymbol{t})$ to the point set $\mathcal{P}$, we will have

$$\begin{aligned} \boldsymbol{A}\boldsymbol{p}_e + \boldsymbol{t} &= \boldsymbol{A}\boldsymbol{p}_a + \boldsymbol{t} + r_1\{(\boldsymbol{A}\boldsymbol{p}_b + \boldsymbol{t}) - (\boldsymbol{A}\boldsymbol{p}_a + \boldsymbol{t})\} \\ &= \boldsymbol{p}_{a'} + r_1(\boldsymbol{p}_{b'} - \boldsymbol{p}_{a'}) = \boldsymbol{p}_{e'} \end{aligned} \tag{1}$$

and

$$\begin{aligned} \boldsymbol{A}\boldsymbol{p}_e + \boldsymbol{t} &= \boldsymbol{A}\boldsymbol{p}_c + \boldsymbol{t} + r_2\{(\boldsymbol{A}\boldsymbol{p}_d + \boldsymbol{t}) - (\boldsymbol{A}\boldsymbol{p}_c + \boldsymbol{t})\} \\ &= \boldsymbol{p}_{c'} + r_2(\boldsymbol{p}_{d'} - \boldsymbol{p}_{c'}) = \boldsymbol{p}_{e'} \end{aligned} \tag{2}$$

where $\boldsymbol{p}_{a'}, \boldsymbol{p}_{b'}, \boldsymbol{p}_{c'}, \boldsymbol{p}_{d'}$ are the correspondences for $\boldsymbol{p}_a, \boldsymbol{p}_b, \boldsymbol{p}_c, \boldsymbol{p}_d$ under the transform $\mathcal{T}(\cdot; \boldsymbol{A}, \boldsymbol{t})$, respectively. It can be easily found that point $\boldsymbol{p}_{e'}$ is the intersection of $\boldsymbol{l}_{a'b'}$ and $\boldsymbol{l}_{c'd'}$, and the system preserves the ratios of r_1 and r_2. □

S1.2 Proof for the constraint of the area ratio between micrographs with different tilted angles

Given a micrograph with tilt angle β_1 and another one with tilt angle β_2, the area of the corresponding plane shapes on these two micrographs have an approximate ratio of $\cos\beta_1/\cos\beta_2$.

Brief proof: By omitting the scale change, the projection model in ET can be described as

$$\begin{pmatrix} u \\ v \end{pmatrix} = \boldsymbol{R}_\gamma \boldsymbol{P} \boldsymbol{R}_\beta \boldsymbol{R}_\alpha \begin{pmatrix} X \\ Y \\ Z \end{pmatrix} + \boldsymbol{t}, \tag{3}$$

where $(X, Y, Z)^T$ is a coordinate representing a spatial point located in the ultrastructure; α represents the pitch angle along the tilt axis; β represents the tilt angle; γ represents the in-plane rotation within the projection plane; $\boldsymbol{t}$ represents the translation of the view; $(u, v)^T$ is the measured projection point; and $\boldsymbol{P}$ denotes the orthogonal projection matrix. Here, the detailed $\boldsymbol{R}_\alpha$, $\boldsymbol{R}_\beta$, $\boldsymbol{P}$ and $\boldsymbol{R}_\gamma$ are defined as:

$$\boldsymbol{R}_\alpha = \begin{pmatrix} 1 & 0 & 0 \\ 0 & \cos\alpha & \sin\alpha \\ 0 & -\sin\alpha & \cos\alpha \end{pmatrix}, \boldsymbol{R}_\beta = \begin{pmatrix} \cos\beta & 0 & -\sin\beta \\ 0 & 1 & 0 \\ \sin\beta & 0 & \cos\beta \end{pmatrix},$$

$$\boldsymbol{P} = \begin{pmatrix} 1 & 0 & 0 \\ 0 & 1 & 0 \end{pmatrix}, \boldsymbol{R}_\gamma = \begin{pmatrix} \cos\gamma & \sin\gamma \\ -\sin\gamma & \cos\gamma \end{pmatrix}.$$

Usually, the pitch angle is very small. By setting the value of α to 0 and simplifying Eq.3, a modified projection model is obtained as

$$\begin{pmatrix} u \\ v \end{pmatrix} = \begin{pmatrix} \cos\gamma & \sin\gamma \\ -\sin\gamma & \cos\gamma \end{pmatrix} \begin{pmatrix} \cos\beta & 0 \\ 0 & 1 \end{pmatrix} \begin{pmatrix} X \\ Y \end{pmatrix} + \boldsymbol{t}, \tag{4}$$

which means that the micrograph with β tilt angle is approximately related to the micrograph with $0°$ by an affine transformation $\mathcal{T}(\cdot; \boldsymbol{A}_\beta, \boldsymbol{t})$, where $\boldsymbol{A}_\beta = \boldsymbol{R}_\gamma \cdot \mathrm{diag}(\cos\beta, 1)$.

According to the area ratio lemma, a shape $\boldsymbol{S}$ on the $0°$ micrograph has a constant area ratio change σ on its transformed micrograph. For the micrograph with β tilt angle, the ratio can be calculated as

$$\sigma = \frac{|\mathcal{T}(\boldsymbol{S})|}{|\boldsymbol{S}|} = |\boldsymbol{A}| = \begin{vmatrix} \cos\gamma & \sin\gamma \\ -\sin\gamma & \cos\gamma \end{vmatrix} \cdot \begin{vmatrix} \cos\beta & 0 \\ 0 & 1 \end{vmatrix} = \cos\beta. \tag{5}$$

Consequently, for the micrographs with tilt angle β_1 and β_2, their area ratio is approximate $(\cos\beta_1|\boldsymbol{S}|)/(\cos\beta_2|\boldsymbol{S}|) = \cos\beta_1/\cos\beta_2$. □

S2 Experimental results

S2.1 Illustration of the datasets

Six real-world datasets are used to evaluate the proposed method. The first dataset is a tilt series that has been used in the previous studies, provided by the Institute of Biophysics, Chinese Academy of Sciences. The remaining five datasets are downloaded from the Caltech ETDB.

The first dataset, Hemocyanin, is a tilt series of vitrified keyhole limpet hemocyanin solution (Fig. S1A). It is a cryo-ET dataset with about 100$\sim$150 fiducial markers embedded in. The tilt series were collected by FEI Titan Krios (300 kV) with a Gatan US4000 camera. The total dose used during data collection was around 8000 e/nm^2. There are 95 images with the tilt ranging from $-70°$ to $70°$ at $1°\sim2°$ intervals ($2K \times 2K$ pixels with 0.4 nm/px).

The second and third datasets, Vibrio1-2, are two cryo-ET datasets of isolated Vibrio cholerae cells (Fig. S1B&C). Vibrio1 and Vibrio2 have about 150$\sim$200 and 200$\sim$250 fiducial markers embedded in the specimens, respectively. Both of the tilt series were collected by FEI Tecnai Polara (F30) (300 kV) with a Gatan K2 camera, operated at 145eV/Å^2 dosage. There are 121 images with the tilt ranging from $-60°$ to $60°$ at $1°$ interval ($4K \times 4K$ pixels with 0.4 nm/px).

The fourth to sixth datasets, Nitrosop1-3, are three cryo-ET datasets of isolated Nitrosopumilus maritimus cells (Fig. S1D-F). Nitrosop1 has about 250$\sim$300 fiducial markers embedded in the specimen, while both Nitrosop2 and Nitrosop3 have about 400$\sim$500 fiducial markers. The Nitrosop1-3 were collected by FEI Tecnai Polara (F30) (300 kV) with a Gatan K2 camera. The Nitrosop1 was operated at 150eV/Å^2 dosage with -10 μm defocus; the Nitrosop2 and Nitrosop3 were operated at 180eV/Å^2 dosage. There are 121 images for Nitrosop1 with the tilt ranging from $-60°$ to $60°$ at $1°$ interval ($4K \times 4K$ pixels with 0.64 nm/px), and 111 images for both Nitrosop2 and Nitrosop3 with the tilt angles ranging from $-55°$ to $55°$ at $1°$ interval ($4K \times 4K$ pixels with 0.49 nm/px).

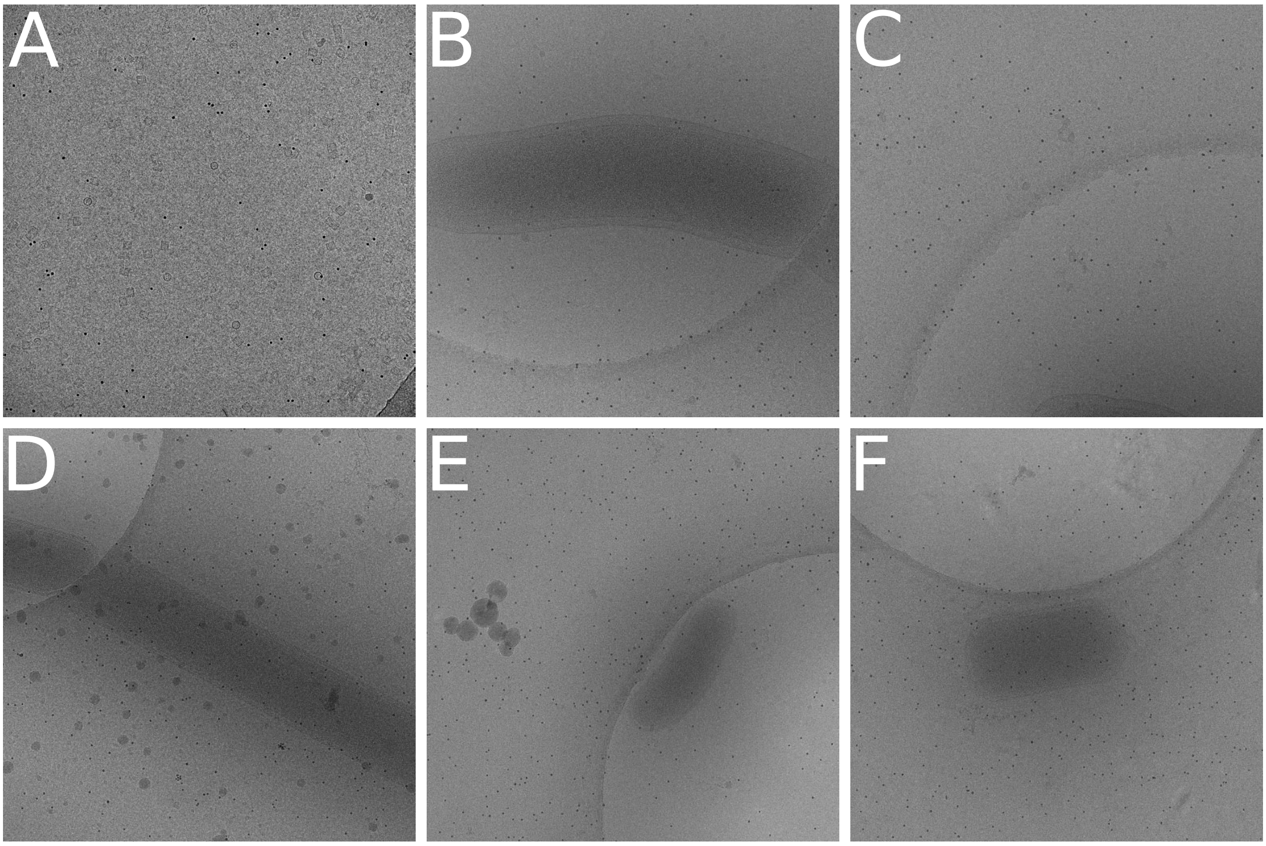

Figure S1: Illustration ($0°$ micrographs) of the test datasets. (A) Hemocyanin, (B) Vibrio1, (C) Vibrio2, (D) Nitrosop1, (E) Nitrosop2 and (F) Nitrosop3.

S2.2 Robustness of the two-stage algorithm under various conditions

In the following, we select the experiment results carried out on micrograph pairs with tilt angles $0°$ and $1°$, and micrograph pairs with tilt angles $0°$ and $45°$ for each dataset as a detailed demonstration.

Hemocyanin:

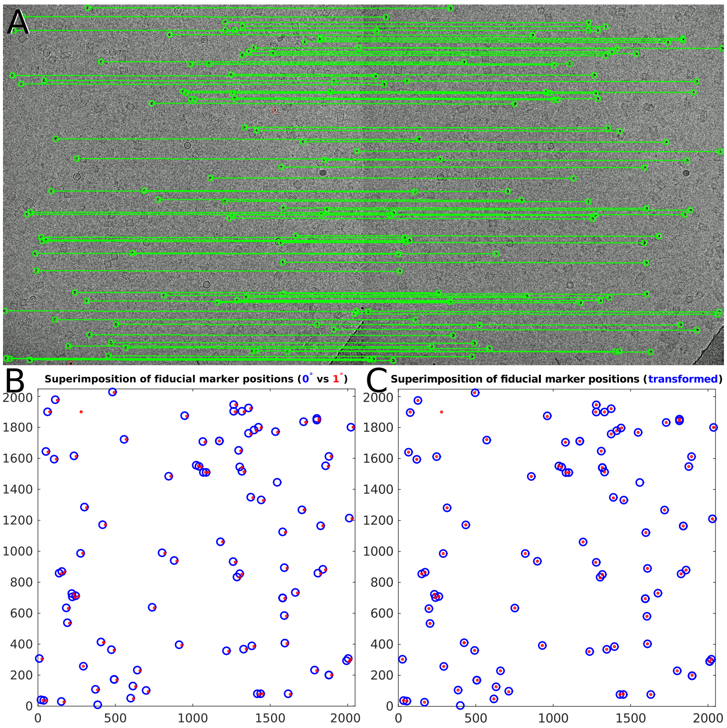

Figure S2: Fiducial marker correspondence determined by the two-stage algorithm for the Hemocyanin dataset. (A) The detected fiducial markers and determined fiducial marker correspondences illustrated on the raw micrographs. (B) Superimposition of fiducial marker positions from the micrographs with 0° and 1° tilt angles. (C) Superimposition of fiducial marker positions after a transformation has been applied to the ones from the 0° tilted micrograph.

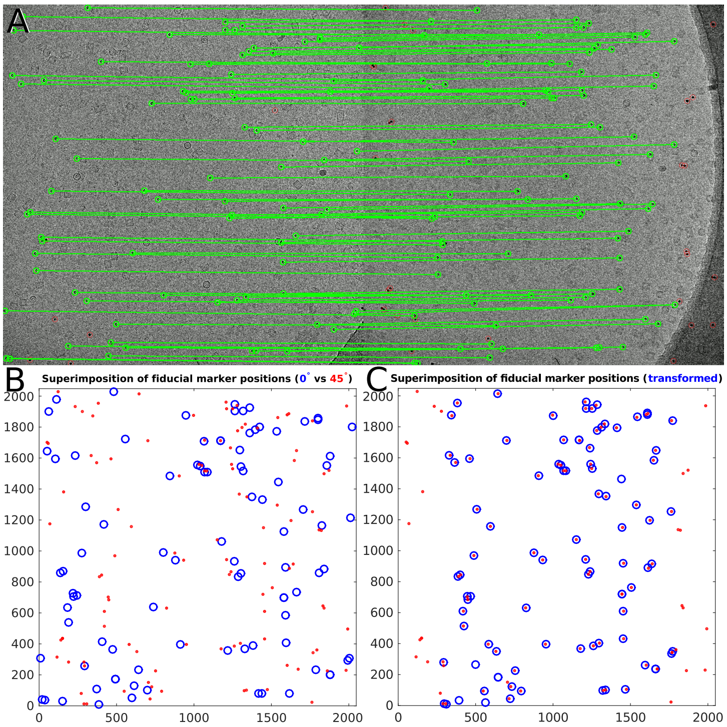

Figure S3: Fiducial marker correspondence determined by the two-stage algorithm for the Hemocyanin dataset. (A) The detected fiducial markers and determined fiducial marker correspondences illustrated on the raw micrographs. (B) Superimposition of fiducial marker positions from the micrographs with 0° and 45° tilt angles. (C) Superimposition of fiducial marker positions after a transformation has been applied to the ones from the 0° tilted micrograph.

Vibrio1:

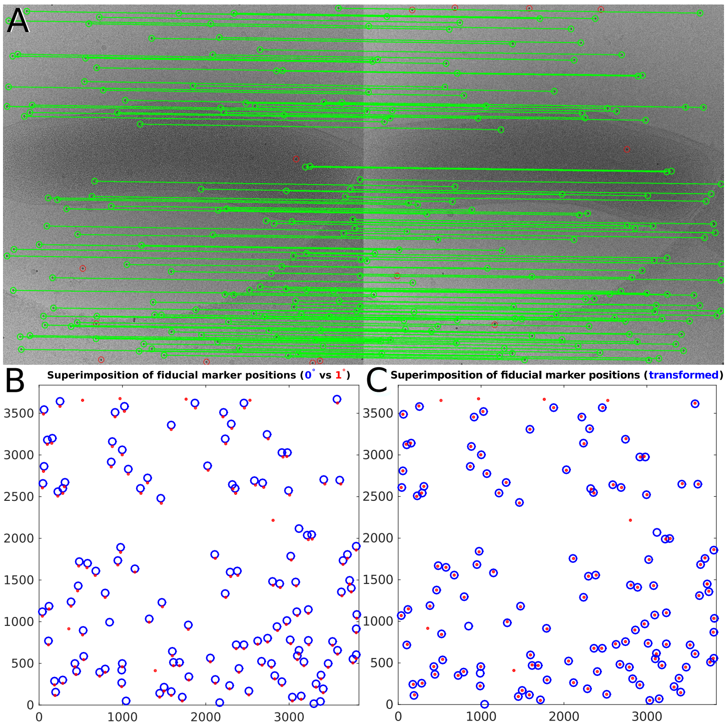

Figure S4: Fiducial marker correspondence determined by the two-stage algorithm for the Vibrio1 dataset. (A) The detected fiducial markers and determined fiducial marker correspondences illustrated on the raw micrographs. (B) Superimposition of fiducial marker positions from the micrographs with 0° and 1° tilt angles. (C) Superimposition of fiducial marker positions after a transformation has been applied to the ones from the 0° tilted micrograph.

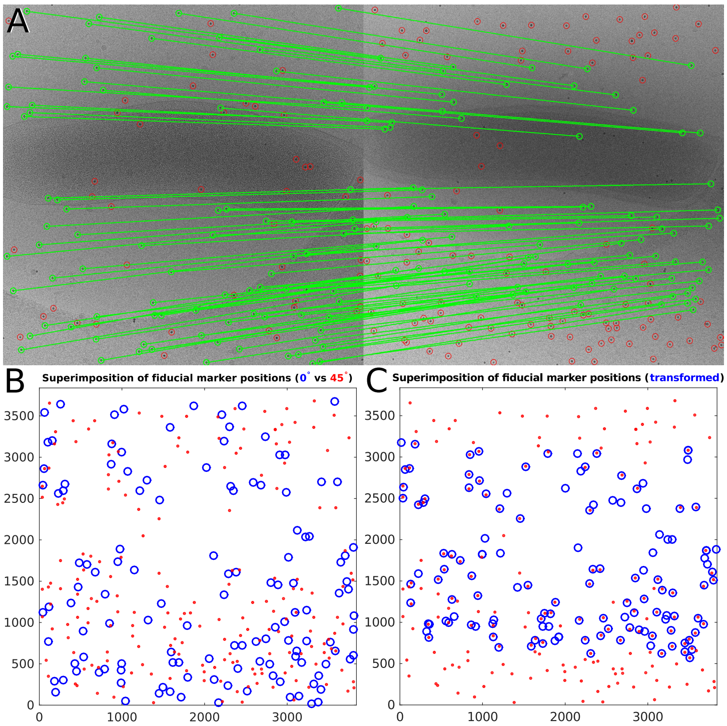

Figure S5: Fiducial marker correspondence determined by the two-stage algorithm for the Vibrio1 dataset. (A) The detected fiducial markers and determined fiducial marker correspondences illustrated on the raw micrographs. (B) Superimposition of fiducial marker positions from the micrographs with 0° and 45° tilt angles. (C) Superimposition of fiducial marker positions after a transformation has been applied to the ones from the 0° tilted micrograph.

Vibrio2:

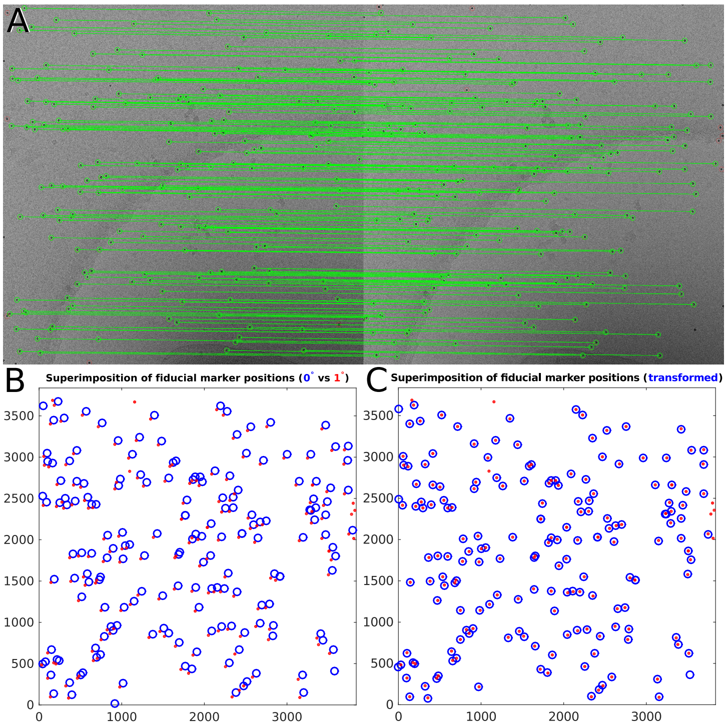

Figure S6: Fiducial marker correspondence determined by the two-stage algorithm for the Vibrio2 dataset. (A) The detected fiducial markers and determined fiducial marker correspondences illustrated on the raw micrographs. (B) Superimposition of fiducial marker positions from the micrographs with 0° and 1° tilt angles. (C) Superimposition of fiducial marker positions after a transformation has been applied to the ones from the 0° tilted micrograph.

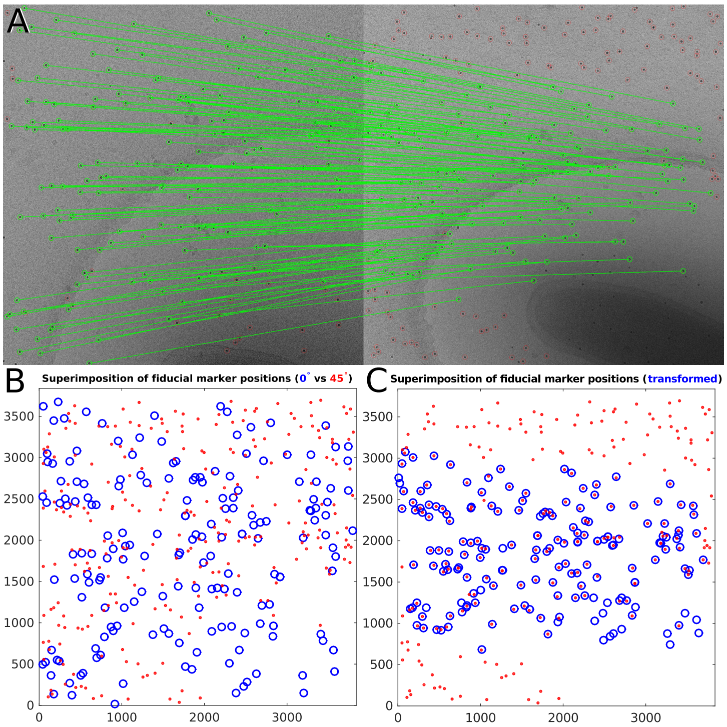

Figure S7: Fiducial marker correspondence determined by the two-stage algorithm for the Vibrio2 dataset. (A) The detected fiducial markers and determined fiducial marker correspondences illustrated on the raw micrographs. (B) Superimposition of fiducial marker positions from the micrographs with 0° and 45° tilt angles. (C) Superimposition of fiducial marker positions after a transformation has been applied to the ones from the 0° tilted micrograph.

Nitrosop1:

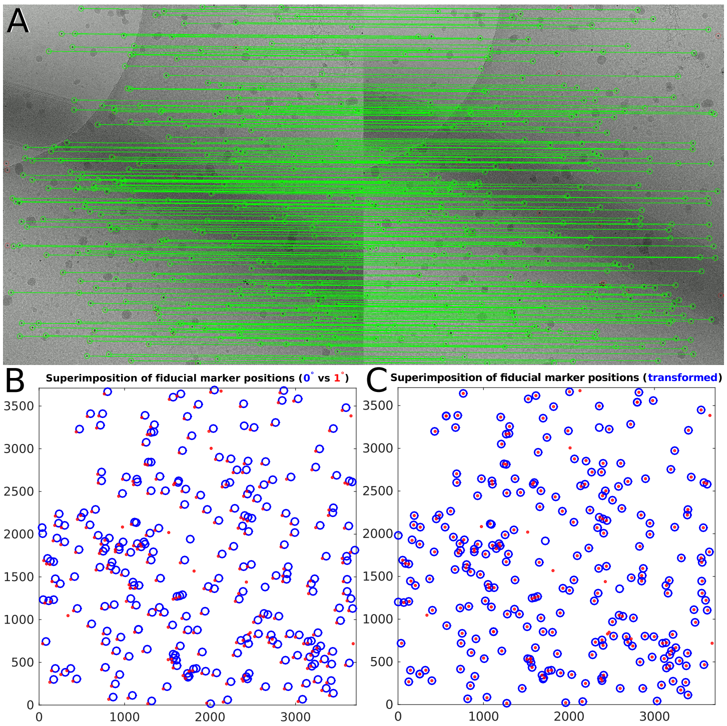

Figure S8: Fiducial marker correspondence determined by the two-stage algorithm for the Nitrosop1 dataset. (A) The detected fiducial markers and determined fiducial marker correspondences illustrated on the raw micrographs. (B) Superimposition of fiducial marker positions from the micrographs with 0° and 1° tilt angles. (C) Superimposition of fiducial marker positions after a transformation has been applied to the ones from the 0° tilted micrograph.

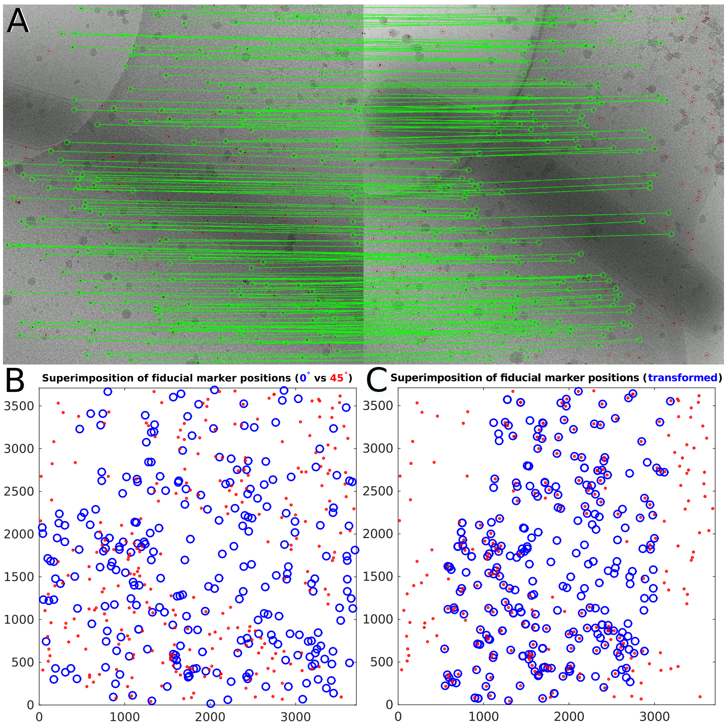

Figure S9: Fiducial marker correspondence determined by the two-stage algorithm for the Nitrosop1 dataset. (A) The detected fiducial markers and determined fiducial marker correspondences illustrated on the raw micrographs. (B) Superimposition of fiducial marker positions from the micrographs with 0° and 45° tilt angles. (C) Superimposition of fiducial marker positions after a transformation has been applied to the ones from the 0° tilted micrograph.

Nitrosop2:

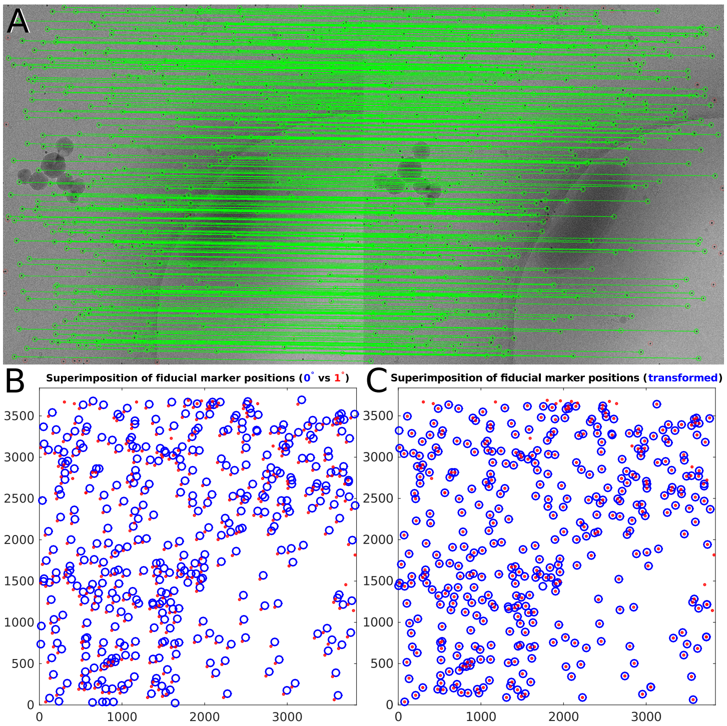

Figure S10: Fiducial marker correspondence determined by the two-stage algorithm for the Nitrosop2 dataset. (A) The detected fiducial markers and determined fiducial marker correspondences illustrated on the raw micrographs. (B) Superimposition of fiducial marker positions from the micrographs with 0° and 1° tilt angles. (C) Superimposition of fiducial marker positions after a transformation has been applied to the ones from the 0° tilted micrograph.

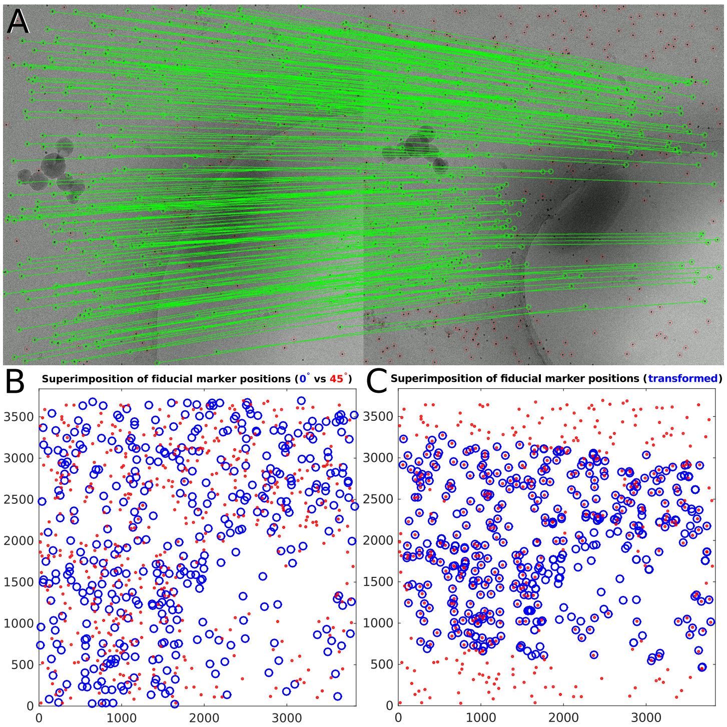

Figure S11: Fiducial marker correspondence determined by the two-stage algorithm for the Nitrosop2 dataset. (A) The detected fiducial markers and determined fiducial marker correspondences illustrated on the raw micrographs. (B) Superimposition of fiducial marker positions from the micrographs with 0° and 45° tilt angles. (C) Superimposition of fiducial marker positions after a transformation has been applied to the ones from the 0° tilted micrograph.

Nitrosop3:

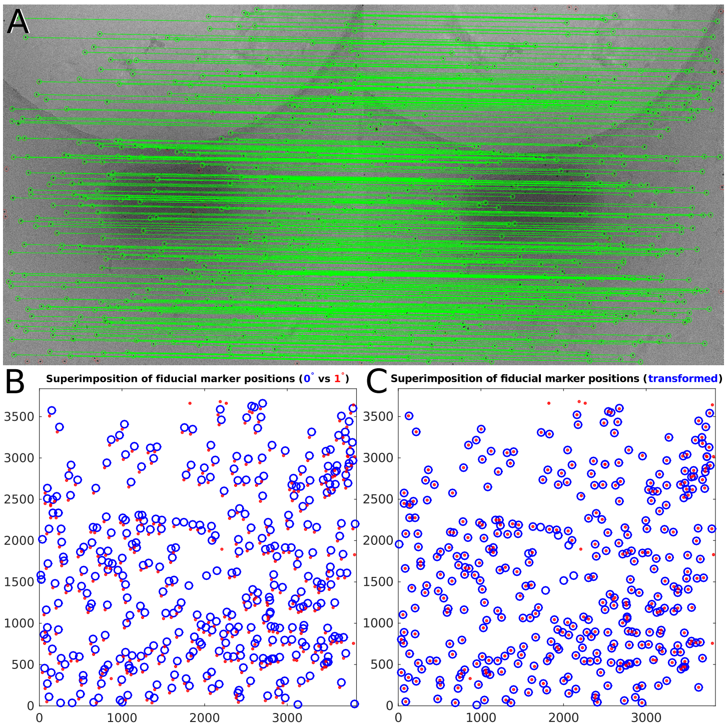

Figure S12: Fiducial marker correspondence determined by the two-stage algorithm for the Nitrosop3 dataset. (A) The detected fiducial markers and determined fiducial marker correspondences illustrated on the raw micrographs. (B) Superimposition of fiducial marker positions from the micrographs with 0° and 1° tilt angles. (C) Superimposition of fiducial marker positions after a transformation has been applied to the ones from the 0° tilted micrograph.

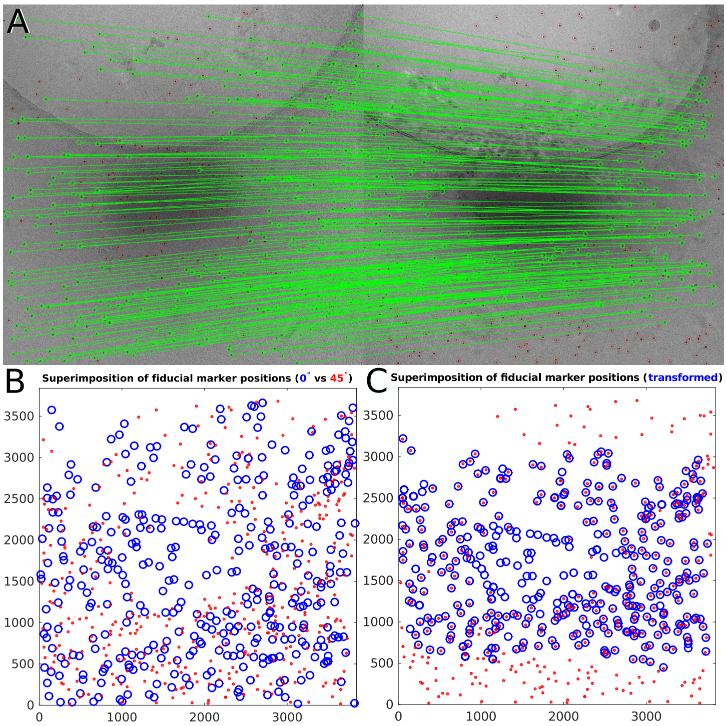

Figure S13: Fiducial marker correspondence determined by the two-stage algorithm for the Nitrosop3 dataset. (A) The detected fiducial markers and determined fiducial marker correspondences illustrated on the raw micrographs. (B) Superimposition of fiducial marker positions from the micrographs with 0° and 45° tilt angles. (C) Superimposition of fiducial marker positions after a transformation has been applied to the ones from the 0° tilted micrograph.

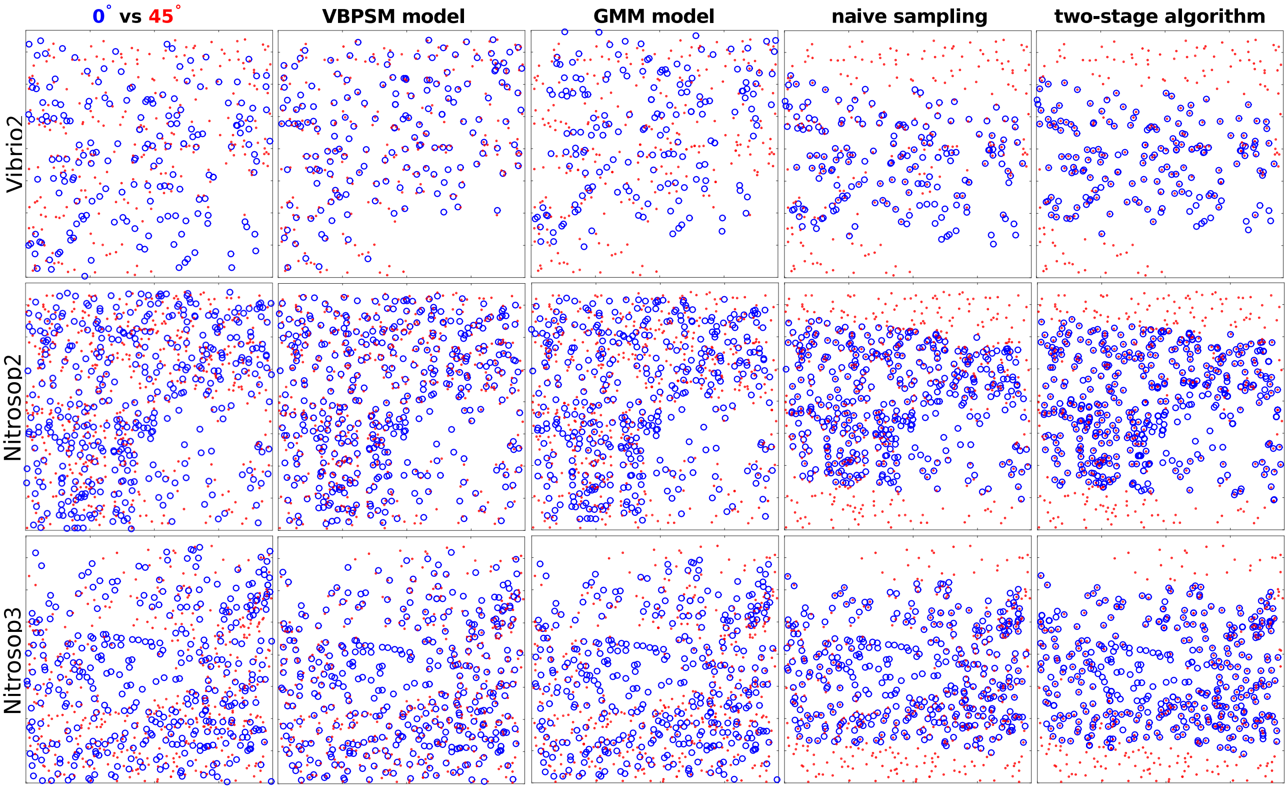

Figure S14: Performance comparison between different fiducial marker tracking methods on the Vibrio2, Nitrosop2 and Nitrosop3 datasets. The 1st column illustrates the superimposition of the raw fiducial marker positions, where the blue 'circle' and red 'dot' denote the fiducial markers extracted from the 0° and 45° tilted micrographs, respectively. The 2nd, 3rd, 4th and 5th columns illustrate the transformed fiducial marker positions of the 0° tilted micrograph solved by the VBPSM model, the GMM model, naive sampling and the two-stage algorithm, respectively.

S2.3 Efficient and effectiveness of the algorithm

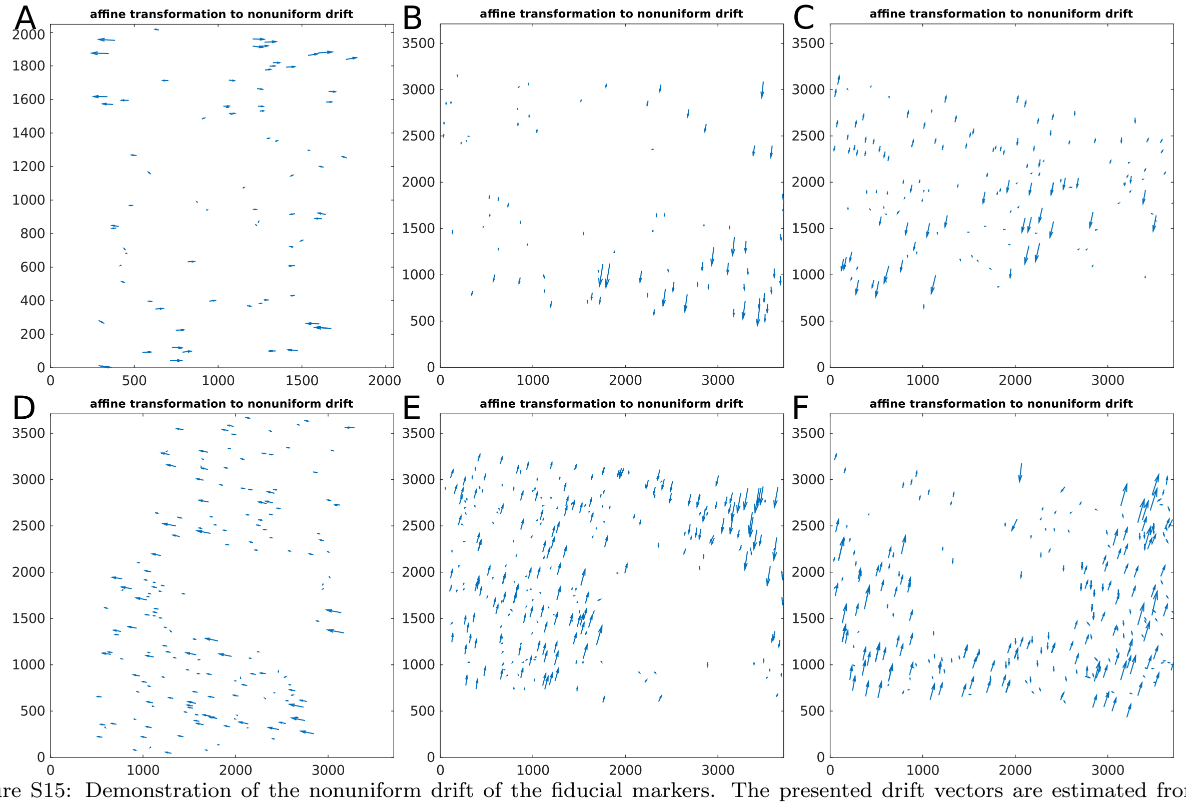

Figure S15: Demonstration of the nonuniform drift of the fiducial markers. The presented drift vectors are estimated from the 0° and 45° tilted micrographs of the datasets. (A) Hemocyanin, (B) Vibrio1, (C) Vibrio2, (D) Nitrosop1, (E) Nitrosop2 and (F) Nitrosop3.
